# RAG-GNN: retrieval-augmented graph neural networks for protein interaction network embeddings

**DOI:** 10.3389/frai.2026.1851917

**Published:** 2026-07-10

**Authors:** Hasi Hays, William J. Richardson

**Affiliations:** Department of Chemical Engineering, University of Arkansas, Fayetteville, AR, United States

**Keywords:** AI in medicine, graph neural network (GNN), network medicine, network modeling, protein interaction networks, retrieval-augmented generation (RAG)

## Abstract

We present RAG-GNN, an end-to-end trainable framework that augments a graph neural network (GNN) encoder for protein interaction networks with a jointly optimized dense retrieval module over a TF-IDF-indexed document corpus, a gated fusion mechanism, and contrastive alignment between node and document representations. The study is positioned as a controlled methodological investigation of whether retrieval augmentation provides measurable benefit beyond a matched GNN-only ablation, rather than as a precision-medicine or therapeutic-target discovery tool. On a cancer signaling case study (379 proteins, 3,498 interactions, 14 pathway categories), RAG-GNN improves functional clustering silhouette from −0.237 ± 0.065 (GNN-only) to −0.144 ± 0.066 (+0.093 ± 0.022 across 10 seeds; ARI +0.021 ± 0.015), while the learned retrieval projection attains mean precision at 10 = 0.242, a 152% relative improvement over a random baseline (0.096). Counterfactual experiments confirm that random and absent retrieval contexts degrade performance, but a shuffled-document control (real documents reassigned to incorrect proteins) performs comparably to proper retrieval, indicating that the gain reflects general biological signal in the aggregate TF-IDF corpus rather than node-specific semantic matching. A heuristic information decomposition with bootstrap confidence intervals shows that topology and retrieval encode overwhelmingly shared information (95.6%), with retrieval contributing primarily by reorganizing this shared signal. Benchmarking against eight embedding methods reveals task-specific complementarity: Topology-focused methods are stronger for link prediction, while retrieval augmentation improves functional clustering within the controlled ablation. A DDR1 subnetwork analysis is reported as confirmatory recovery of biology already present in the input corpus, not as prospective discovery.

## 1 Introduction

Computational analysis of biological systems increasingly relies on integrating heterogeneous data sources, including genomic sequences, protein interaction networks, metabolic pathways, and the biomedical literature (Barabási et al., 2011; Zitnik et al., 2018). Network-based representations provide a system-level framework in which molecular components are encoded as nodes and their interactions as edges (Ideker and Krogan, 2012; Gysi et al., 2021), and the central premise of this view is that node-level function emerges from patterns of interaction rather than from any single component in isolation (Menche et al., 2015). The past decade has witnessed rapid development of network embedding methods that learn low-dimensional vector representations of nodes while preserving structural properties. Random walk-based approaches such as DeepWalk (Perozzi et al., 2014) and Node2Vec (Grover and Leskovec, 2016) generate node sequences through stochastic walks and apply skip-gram models to learn embeddings that capture neighborhood co-occurrence patterns. LINE (Tang et al., 2015) explicitly optimizes for first-order (direct connection) and second-order (shared neighborhood) proximity preservation. Spectral methods (Belkin and Niyogi, 2002) derive embeddings from eigenvectors of the graph Laplacian, providing theoretical guarantees for preserving global structure.

Graph neural networks (GNNs) have emerged as the dominant paradigm for learning on graph-structured data (Gilmer et al., 2017). GCN (Kipf and Welling, 2017) implements spectral convolutions through neighborhood aggregation, while GraphSAGE (Hamilton et al., 2017) enables inductive learning through sampling-based aggregation. Graph attention networks (GAT) (Veličković et al., 2018) introduce attention mechanisms to weight neighbor contributions adaptively. These methods achieve remarkable performance on structural prediction tasks (link prediction, node classification based on network position, and community detection) because they directly encode the topological features that determine these outcomes. However, a fundamental limitation emerges when network embeddings are applied to *functional* prediction tasks. Predicting protein function or grouping proteins by biological role requires information about biological mechanisms that extends beyond network topology. Two proteins may occupy similar network positions yet perform entirely different cellular functions; conversely, functionally related proteins may reside in distant network neighborhoods. This structure-function gap represents a methodological challenge: Network topology is necessary but, on its own, insufficient for functional interpretation of node-level biology. Mechanistic information about protein function is distributed across millions of publications and curated pathway resources, encompassing details of molecular mechanism, tissue-specific expression patterns, post-translational modifications, genetic variant effects, and pairwise interactions. Crucially, much of this information is largely absent from the network structure itself: An edge between two proteins indicates a physical or functional interaction but reveals little about the molecular consequences of that interaction. Traditional approaches to incorporating external knowledge rely on knowledge graphs with fixed schemas (Bordes et al., 2013), which require explicit entity extraction and relationship annotation. While effective for structured knowledge, these approaches cannot easily accommodate the nuanced, context-dependent information in unstructured text. The exponential growth of biomedical literature (over 1.5 million PubMed articles annually) makes manual curation increasingly intractable, creating a widening gap between published knowledge and computationally accessible information.

Retrieval-augmented generation (RAG) architectures provide a framework for dynamically integrating external knowledge into predictive systems (Lewis et al., 2020; Gao et al., 2023; Borgeaud et al., 2022). RAG systems couple neural retrievers that identify relevant documents from large corpora with models that synthesize retrieved information into predictions. Unlike knowledge graphs with fixed schemas, RAG systems access unstructured text, adapt to new information without retraining, and provide interpretable evidence through retrieved documents. The success of RAG in natural language processing, where retrieved context dramatically improves factual accuracy and reduces hallucination, suggests potential for similar benefits in computational biology. Applying RAG to biological network modeling requires addressing domain-specific challenges. First, the retrieval mechanism must identify documents relevant to specific molecular entities within massive biomedical corpora. Second, retrieved information must be fused with network-derived representations in a manner that preserves both topological and semantic structure. Third, the joint system must be validated to ensure that retrieved knowledge provides genuinely novel information beyond what network topology alone encodes rather than simply increasing model capacity.

The central challenge lies in creating embedding spaces that coherently represent both network topology and semantic biological knowledge. Graph neural networks learn node representations through message-passing operations (Kipf and Welling, 2017; Veličković et al., 2018), while transformer architectures encode textual information through self-attention mechanisms (Vaswani et al., 2017; Devlin et al., 2019). Recent advances in foundation models for biology have demonstrated the power of large-scale pretraining on protein sequences (Rives et al., 2021; Lin et al., 2023), gene expression data (Theodoris et al., 2023; Cui et al., 2024), and molecular structures (Zhou et al., 2023). Integrating these paradigms requires careful formulation to ensure structural and semantic information reinforce rather than interfere with each other. A critical empirical question motivates this work: *Do topology-only and retrieval-augmented embeddings excel at the same tasks, or do they exhibit complementary strengths?* If the latter, understanding when each approach is most appropriate becomes essential for method selection in computational biology. We address this question through comprehensive benchmarking across multiple prediction tasks, information-theoretic decomposition of predictive contributions, and counterfactual experiments that isolate retrieval effects.

**Figure 1:**
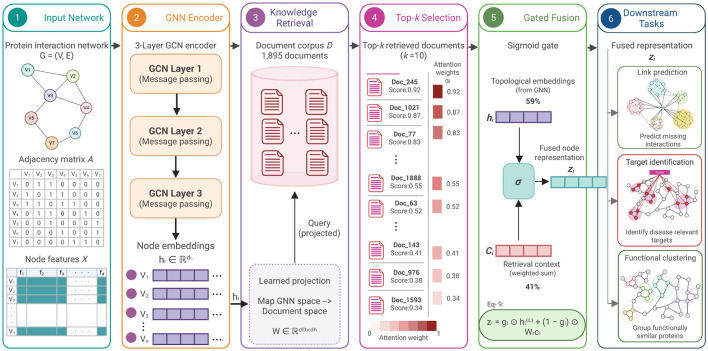
RAG-GNN framework: End-to-end architecture overview for the controlled study. The framework comprises six sequential stages used in the controlled comparison against a matched GNN-only ablation. (1) Input Network: A protein interaction network *G* = (*V, E*) is represented by its adjacency matrix *A* and node feature matrix *X* (three topological features per node). (2) GNN Encoder: A 3-layer GCN performs iterative message passing over the graph, producing node embeddings ${\text{h}}_{i}\in {\mathbb{R}}^{d_{h}}$ that capture local and higher-order topological structure. (3) Document Retrieval: Each node embedding is projected into a document space via a learned projection matrix $W\in {\mathbb{R}}^{d_{D}\times d_{h}}$, which queries a TF-IDF-indexed corpus D of 1,895 biomedical documents. (4) Top-*k* Selection: The *k* = 10 highest-scoring documents per node are selected, and attention weights α_*i*_ produce a weighted retrieval context vector c_*i*_. (5) Gated Fusion: A sigmoid gate *g*_*i*_ = σ(*W*_*g*_[h_*i*_||c_*i*_] + *b*_*g*_) learns per-node weighting between topology embeddings (≈ 59%) and retrieval context (≈ 41%), producing fused representations z_*i*_ = *g*_*i*_ ⊙ h_*i*_ + (1 − *g*_*i*_) ⊙ c_*i*_. (6) Downstream evaluation: The fused embeddings z_*i*_ are evaluated on functional clustering, link prediction, and node classification within the same controlled protocol used for all baselines. Created in BioRender: Hays, H. (2026). https://BioRender.com/fl4ty7d. Agreement number: MX29PM7RGB.

This manuscript develops a controlled methodological study of retrieval augmentation for graph neural network embeddings of protein interaction networks, unifying GNN-based topology encoding with RAG-based document retrieval through a jointly optimized embedding space and quantifying its behavior relative to a matched GNN-only ablation (Figure 1). Our contributions are as follows:

- *Architecture and training objective*. A jointly trained GNN encoder, learned dense-retrieval projection over a TF-IDF-indexed corpus, gated fusion, and a three-component objective (task loss, retrieval ranking loss, contrastive alignment), with operator-level formulation in the main text and the supporting Lipschitz / Rademacher-style propositions deferred to Appendix B as standard background rather than novel theoretical results.
- *Controlled benchmark*. A matched-architecture comparison of RAG-GNN against its own GNN-only ablation, together with seven additional embedding baselines (DeepWalk, Node2Vec, LINE, Spectral, GCN, GAT, GraphSAGE, and raw features), evaluated across functional clustering, link prediction, and node classification on 10 random seeds with standard deviations and confidence intervals, isolating the contribution of retrieval rather than that of additional model capacity.
- *Information-theoretic and counterfactual diagnostics*. A heuristic mutual-information decomposition with 200 bootstrap resamples and 95% confidence intervals quantifying the shared, unique, and synergistic information carried by topology and retrieval (95.6% shared, 0.1% / 6.2% unique, and 0.4% synergy), and counterfactual experiments that compare proper retrieval to random, absent, adversarial, and shuffled-document controls. The shuffled-document control performs comparably to proper retrieval and is reported as a substantive negative finding, indicating that the gain reflects general TF-IDF biological signal rather than node-specific semantic matching.
- *Case study with explicit scope*. A cancer signaling network (379 proteins, 3,498 interactions, 14 pathway categories) on which retrieval integration consistently improves functional clustering over the GNN-only ablation (silhouette +0.093 ± 0.022, ARI +0.021 ± 0.015 across all seeds; learned retrieval precision at 10 = 0.242, +152% over random). A DDR1 subnetwork analysis is reported as confirmatory recovery of biology already represented in the input corpus, not as a precision-medicine or target-discovery contribution; the corresponding temporal therapeutic-target evaluation is reported transparently as inconclusive (AUROC 0.450 ± 0.088 on three post-2020 test targets).

Within this controlled setting, topology-only and retrieval-augmented embeddings exhibit complementary behavior on the cancer signaling network: Structural prediction tasks (link prediction) are served effectively by network topology alone, while functional clustering benefits from the addition of retrieved documents over a matched GNN-only baseline. We treat this as evidence of a method-level effect on one PPI-scale case study and explicitly defer claims about precision medicine, target discovery, and clinical deployment to future work on larger networks and richer document encoders (Section 10). Beyond the scope of this controlled comparison, the joint document–network architecture is intended as methodological groundwork for AI-in-medicine applications that integrate biomedical document evidence with protein interaction networks; the present PPI-scale findings are reported as such groundwork, and empirical validation of any specific downstream medical application is explicitly deferred to future work.

## 2 Mathematical foundations of RAG embeddings

**Figure 2:**
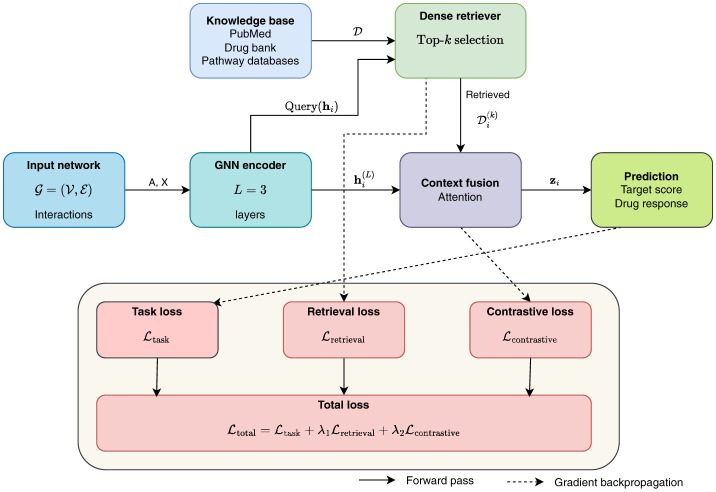
RAG-GNN architecture for retrieval-augmented network embedding. The complete system integrates network topology encoding, document retrieval, and context fusion through six main components. The forward pass (solid arrows) begins with the input network G=(A,X) representing molecular interactions and node features. The GNN encoder applies *L* layers of message passing to produce structural node embeddings ${\text{h}}_{i}^{\left(L\right)}$ that capture network topology (Equation 1). These embeddings serve dual purposes: (1) querying the document corpus through the dense retriever to identify top-*k* relevant documents $D_{i}^{\left(k\right)}$ from the document corpus (Equation 5) and (2) providing structural context for fusion. Retrieved documents are aggregated with attention weighting and fused with structural embeddings ${\text{h}}_{i}^{\left(L\right)}$ through the context fusion module to produce final node representations z_*i*_ (Equation 8). These representations can be passed to a task-specific prediction head. In the present case study, the prediction head is instantiated only for functional clustering and link prediction on the 379-protein cancer signaling network and for the temporal therapeutic-target evaluation reported as inconclusive in Section 4.2; therapeutic-target scoring and drug-response prediction heads are illustrative downstream task formulations (Section 5) and are not empirically evaluated here. The training objective (bottom) jointly optimizes three components through gradient backpropagation (dashed arrows): task-specific loss $L_{\text{task}}$ for prediction accuracy, retrieval quality loss $L_{\text{retrieval}}$ ensuring relevant document selection, and contrastive embedding alignment loss $L_{\text{contrastive}}$ coordinating node and document representations in shared semantic space (Equation 10). The multi-objective formulation $L_{\text{total}}=L_{\text{task}}+\lambda_{1}L_{\text{retrieval}}+\lambda_{2}L_{\text{contrastive}}$ enables end-to-end learning where retrieval and embedding components are optimized to support downstream prediction tasks. Curriculum learning stages the training process to ensure stable convergence and effective coordination between network encoding, document retrieval, and context fusion mechanisms.

The theoretical development of RAG-enhanced network modeling requires careful formalization of how biological networks, document corpus, and embedding spaces interact. Figure 2 illustrates the complete RAG-GNN architecture integrating network topology encoding, document retrieval, and context fusion. We begin by establishing notation and mathematical structures and then derive the core embedding mechanisms that enable joint representation learning.

### 2.1 Network topology encoding

Consider a biological network G=(V,E) representing molecular interactions, where the vertex set $V=\left\{v_{1},v_{2},\ldots ,v_{\left|V\right|}\right\}$ contains molecular entities and the edge set E⊆V×V encodes functional relationships. In protein–protein interaction networks, vertices represent individual proteins and edges denote physical binding, regulatory interactions, or pathway co-membership. For metabolic networks, vertices are metabolites or enzymes, while edges represent biochemical transformations. Each node $v_{i}\in V$ possesses intrinsic feature vector ${\text{x}}_{i}\in {\mathbb{R}}^{d_{0}}$ encoding properties such as amino acid sequence embeddings, gene expression levels, protein abundance measurements, or physicochemical characteristics including molecular weight, hydrophobicity, and charge distribution.

The network topology is encoded through an adjacency matrix $\text{A}\in {\left\{0,1\right\}}^{\left|V|\times |V\right|}$ where *A*_*ij*_ = 1 if $\left(v_{i},v_{j}\right)\in E$ and *A*_*ij*_ = 0 otherwise. For weighted networks representing interaction confidence or regulatory strength, we extend to $\text{A}\in {\mathbb{R}}_{+}^{\left|V|\times |V\right|}$ with edge weights derived from experimental evidence, co-expression correlation, or literature support. The degree matrix **D** is diagonal with $D_{ii}=\underset{j}{\sum }A_{ij}$, enabling normalized representations that account for node connectivity.

A graph neural network encoder $f_{\text{GNN}}:{\mathbb{R}}^{|V|\times d_{0}}\to {\mathbb{R}}^{|V|\times d_{h}}$ maps initial node features to latent representations through *L* layers of message-passing operations. The update rule at layer *k* for node *v*_*i*_ aggregates information from neighboring nodes weighted by normalized connectivity:


$$\begin{array}{l} {\text{h}}_{i}^{\left(k+1\right)}=\sigma \left({\text{W}}^{\left(k\right)}{\text{h}}_{i}^{\left(k\right)}+\underset{j\in N\left(i\right)}{\sum }\frac{1}{\sqrt{\left|N\left(i\right)\|N\left(j\right)\right|}}{\text{h}}_{j}^{\left(k\right)}\right) \end{array}$$ (1)

where ${\text{h}}_{i}^{\left(k\right)}\in {\mathbb{R}}^{d_{h}}$ denotes the hidden representation of node *v*_*i*_ at layer *k*, with initialization ${\text{h}}_{i}^{\left(0\right)}={\text{x}}_{i}$. The neighborhood set $N\left(i\right)=\left\{j:A_{ij}>0\right\}$ contains nodes directly connected to *v*_*i*_. Learnable weight matrices ${\text{W}}^{\left(k\right)}\in {\mathbb{R}}^{d_{h}\times d_{h}}$ transform representations, and σ(·) applies non-linear activation (typically ReLU or ELU) element-wise. The symmetric normalization factor $1/\sqrt{\left|N\left(i\right)\|N\left(j\right)\right|}$ ensures numerical stability across nodes with varying degrees, preventing over-representation of high-degree hub nodes.

This message-passing framework implements a spectral graph convolution that can be interpreted as diffusion of node features across network edges. After *L* layers, node *v*_*i*_ has aggregated information from its *L*-hop neighborhood, enabling representations to capture both local motifs and global structural patterns. The choice of *L* represents a trade-off: Small *L* limits receptive field size, while large *L* risks over-smoothing where all node representations converge to similar values.

### 2.2 Document retrieval mechanism

Let $D=\left\{d_{1},d_{2},\ldots ,d_{N}\right\}$ represent a corpus of biological knowledge documents, where each document *d*_*j*_ contains structured or unstructured information about molecular functions, pathway memberships, disease associations, drug interactions, or phenotypic effects. Documents may be PubMed abstracts, Gene Ontology annotations, KEGG pathway descriptions, DrugBank entries, or clinical trial summaries. The corpus size *N* typically ranges from 10^5^ to 10^7^ depending on the domain scope.

We define a retrieval function $R:V\times D\to {\mathbb{R}}^{+}$ that scores the relevance of document *d*_*j*_ to node *v*_*i*_ based on semantic similarity in a learned embedding space:


$$\begin{array}{l} R\left(v_{i},d_{j}\right)=\text{sim}\left(E_{\text{node}}\left(v_{i}\right),E_{\text{doc}}\left(d_{j}\right)\right)\cdot Q\left(d_{j}\right) \end{array}$$ (2)

The node embedding function $E_{\text{node}}:V\to {\mathbb{R}}^{d_{\text{doc}}}$ maps biological entities to a semantic vector space matching the document embedding dimension *d*_doc_. This embedding is derived from node features and network context through a learned projection applied to GNN outputs: in our implementation, a two-layer MLP $E_{\text{node}}\left(v_{i}\right)=f_{\text{proj}}\left({\text{h}}_{i}^{\left(L\right)}\right)$ with $f_{\text{proj}}:{\mathbb{R}}^{d_{h}}\to {\mathbb{R}}^{d_{h}}\to {\mathbb{R}}^{d_{\text{doc}}}$ using GELU activation, where *d*_*h*_ = 128 and *d*_doc_ = 64.

The document embedding function $E_{\text{doc}}:D\to {\mathbb{R}}^{d_{\text{doc}}}$ maps textual content to the same semantic space. In the general framework, *E*_doc_ can be instantiated using pre-trained biomedical language models such as BioBERT or PubMedBERT (Devlin et al., 2019; Gu et al., 2022), fine-tuned on the retrieval task, where document embeddings are computed as *E*_doc_(*d*_*j*_) = mean-pool(BERT(tokenize(*d*_*j*_))). In the current implementation, we use TF-IDF representations (256 features, unigrams and bigrams) followed by truncated SVD for dimensionality reduction to *d*_doc_ = 64 (see Appendix Subsection A.3 for details), which provides a computationally efficient baseline text encoder that isolates the contribution of document retrieval from the choice of text encoder. Replacing TF-IDF with pre-trained biomedical language models represents a natural extension expected to further improve retrieval precision.

The document quality function Q:D→[0,1] weights documents based on evidence level, publication quality, and experimental rigor. In the general framework, quality scores can be computed as a weighted combination of objective metrics:


$$\begin{array}{r} Q\left(d_{j}\right)=w_{1}\cdot \text{study\_type}\left(d_{j}\right)\\ +w_{2}\cdot \text{citation\_impact}\left(d_{j}\right)\\ +w_{3}\cdot \text{journal\_quality}\left(d_{j}\right) \end{array}$$ (3)

where study type assigns weights based on evidence hierarchy (e.g., randomized controlled trials 1.0, prospective cohorts 0.8), citation impact computes the time-adjusted citation percentile, and journal quality uses normalized journal rank. In the current case study implementation, which uses curated mechanistic annotation templates rather than heterogeneous literature, *Q*(*d*_*j*_) = 1 uniformly, all documents are of equal quality by construction. Quality-weighted retrieval becomes relevant when scaling to real biomedical literature corpora with heterogeneous evidence levels, representing a natural extension for deployment scenarios.

The similarity function sim(·, ·) quantifies semantic proximity. We employ scaled dot-product similarity:


$$\begin{array}{l} \text{sim}\left(\text{u},\text{v}\right)=\frac{{\text{u}}^{⊤}\text{v}}{\sqrt{d_{\text{doc}}}} \end{array}$$ (4)

The scaling by $\sqrt{d_{\text{doc}}}$ prevents saturation of downstream softmax operations for high-dimensional embeddings. Alternative formulations include cosine similarity **u**^⊤^**v**/(||**u**||||**v**||) or learned bilinear similarity ${\text{u}}^{⊤}{\text{W}}_{s}\text{v}$ with trainable **W**_*s*_.

For a given node *v*_*i*_, retrieval identifies the top-*k* most relevant documents:


$$\begin{array}{l} D_{i}^{\left(k\right)}=\underset{S\subset D,|S|=k}{\arg\max}\underset{d_{j}\in S}{\sum }R\left(v_{i},d_{j}\right) \end{array}$$ (5)

The hyperparameter *k* controls the breadth of retrieved context. Small *k* (3–5) provides focused information but may miss relevant details. Large *k* (20–50) increases coverage but introduces noise and computational cost. In practice, *k* is tuned via validation set performance on downstream tasks.

### 2.3 Joint embedding architecture

The RAG embedding framework integrates network topology and retrieved knowledge through a multi-stage fusion mechanism. After retrieving documents $D_{i}^{\left(k\right)}$ for node *v*_*i*_, we construct a contextualized knowledge vector that aggregates semantic information from retrieved sources.

Let ${\text{c}}_{i}\in {\mathbb{R}}^{d_{c}}$ represent the contextualized knowledge vector for node *v*_*i*_, computed as a weighted aggregation of retrieved document embeddings with attention-based importance weighting:


$$\begin{array}{l} {\text{c}}_{i}=\underset{d_{j}\in D_{i}^{\left(k\right)}}{\sum }\alpha_{ij}E_{\text{doc}}\left(d_{j}\right) \end{array}$$ (6)

The attention weights α_*ij*_ are derived through a softmax-normalized scoring function that prioritizes highly relevant documents:


$$\begin{array}{l} \alpha_{ij}=\frac{\exp\left(R\left(v_{i},d_{j}\right)/\tau \right)}{\sum_{d_{\ell }\in D_{i}^{\left(k\right)}}\exp\left(R\left(v_{i},d_{\ell }\right)/\tau \right)} \end{array}$$ (7)

The temperature parameter τ controls attention sharpness: Small τ concentrates weight on the single most relevant document (hard attention), while large τ distributes weight more uniformly (soft attention). Typical values range from 0.1 to 1.0.

The final node representation ${\text{z}}_{i}\in {\mathbb{R}}^{d_{z}}$ combines structural information from GNN encoding with semantic context from retrieved documents through a learned fusion function. We employ concatenation followed by linear projection:


$$\begin{array}{l} {\text{z}}_{i}=f_{\text{fusion}}\left({\text{h}}_{i}^{\left(L\right)},{\text{c}}_{i}\right)={\text{W}}_{f}\left[{\text{h}}_{i}^{\left(L\right)}\|{\text{c}}_{i}\right]+{\text{b}}_{f} \end{array}$$ (8)

where [·||·] denotes concatenation, ${\text{W}}_{f}\in {\mathbb{R}}^{d_{z}\times \left(d_{h}+d_{c}\right)}$ is a learnable weight matrix, and ${\text{b}}_{f}\in {\mathbb{R}}^{d_{z}}$ is a bias vector. Alternative fusion strategies include gated mechanisms where the model learns to weight structural vs. semantic information:


$$\begin{array}{l} {\text{z}}_{i}=g_{i}⊙{\text{h}}_{i}^{\left(L\right)}+\left(1-g_{i}\right)⊙{\text{W}}_{r}{\text{c}}_{i} \end{array}$$ (9)

where ${\text{W}}_{r}\in {\mathbb{R}}^{d_{h}\times d_{\text{doc}}}$ projects the retrieval context to match the GNN embedding dimension, and gate values $g_{i}=\sigma \left({\text{W}}_{g}\left[{\text{h}}_{i}^{\left(L\right)}\|{\text{c}}_{i}\right]+{\text{b}}_{g}\right)$ are learned from data.

## 3 Optimization framework

Training the RAG embedding system requires simultaneous optimization of multiple interrelated objectives. The GNN encoder must learn representations that capture network topology, the retrieval mechanism must identify relevant documents, and the fusion module must effectively integrate both information sources. We develop a unified optimization framework that jointly trains all components end-to-end.

### 3.1 Joint training objective

The complete training objective is a weighted combination of task-specific prediction loss, retrieval quality loss, and contrastive embedding alignment loss:


$$\begin{array}{l} L_{\text{total}}=L_{\text{task}}+\lambda_{1}L_{\text{retrieval}}+\lambda_{2}L_{\text{contrastive}} \end{array}$$ (10)

The hyperparameters $\lambda_{1},\lambda_{2}\in {\mathbb{R}}^{+}$ control the relative importance of auxiliary objectives. These are typically set through validation set tuning, with common values λ_1_ ∈ [0.1, 1.0] and λ_2_ ∈ [0.1, 0.5]. The multi-objective formulation ensures that retrieval and embedding alignment support rather than detract from primary task performance.

### 3.2 Task-specific loss

For the downstream task formulations on which RAG-GNN embeddings can be used (identifying disease-relevant nodes, link prediction, target prioritization, or response forecasting), the embedding architecture supports a generic prediction loss over node pairs or node–target pairs. In the present case study, we instantiate this loss only as link prediction over the 379-protein cancer signaling network and as the temporal therapeutic-target prediction reported in Section 4.2; precision-medicine and drug-response formulations are described in Section 5 only as illustrative downstream task formulations and are not empirically evaluated here.

Link prediction aims to infer the probability of an edge between nodes *v*_*i*_ and *v*_*j*_ based on their learned representations. The prediction score is computed as follows:


$$\begin{array}{l} s_{ij}=\sigma \left({\text{z}}_{i}^{⊤}{\text{z}}_{j}\right) \end{array}$$ (11)

where σ(·) is the logistic sigmoid function mapping scores to [0, 1] probabilities. The task loss employs binary cross-entropy over positive (observed) edges $E^{+}$ and negative (unobserved) edges $E^{-}$:


$$\begin{array}{l} L_{\text{task}}=-\underset{\left(i,j\right)\in E^{+}}{\sum }\log\sigma \left({\text{z}}_{i}^{⊤}{\text{z}}_{j}\right)-\underset{\left(i,j\right)\in E^{-}}{\sum }\log\left(1-\sigma \left({\text{z}}_{i}^{⊤}{\text{z}}_{j}\right)\right) \end{array}$$ (12)

Negative edges are sampled uniformly from V×V\E with cardinality $\left|E^{-}|=r|E^{+}\right|$ where *r* ≥ 1 controls the negative sampling ratio. Typical values *r* ∈ [1, 5] balance computational cost with sufficient negative signal.

For drug response prediction tasks, the loss extends to regression objectives predicting continuous efficacy scores or toxicity measures:


$$\begin{array}{l} L_{\text{response}}=\underset{\left(i,d,y\right)\in T}{\sum }{\left({\text{z}}_{i}^{⊤}{\text{z}}_{d}-y\right)}^{2} \end{array}$$ (13)

where T contains tuples of protein node *i*, drug compound *d*, and response value *y*.

### 3.3 Retrieval quality loss

To ensure the retrieval mechanism identifies genuinely relevant documents rather than spuriously similar text, we employ a ranking loss based on relevance judgments. Let $D_{i}^{+}\subset D$ denote the set of ground-truth relevant documents for node *v*_*i*_, established through manual curation or weak supervision from co-occurrence in annotated databases.

The retrieval loss encourages relevant documents to have higher scores than irrelevant documents with a margin γ:


$$\begin{array}{r} L_{\text{retrieval}}=\underset{v_{i}\in V}{\sum }\underset{d_{j}\in D_{i}^{+}}{\sum }\underset{d_{k}\in D_{i}^{-}}{\sum }\\ \max\left(0,\gamma +R\left(v_{i},d_{k}\right)-R\left(v_{i},d_{j}\right)\right) \end{array}$$ (14)

where $D_{i}^{-}=D\backslash D_{i}^{+}$ contains negative (irrelevant) documents. For computational tractability, we sample a subset of negative documents per positive example rather than evaluating all pairs. The margin γ is typically set to 0.1–0.5, enforcing a minimum separation between positive and negative scores.

An alternative formulation uses the softmax-based cross-entropy loss treating retrieval as a classification task:


$$\begin{array}{l} L_{\text{retrieval}}^{\text{CE}}=-\underset{v_{i}\in V}{\sum }\underset{d_{j}\in D_{i}^{+}}{\sum }\log\frac{\exp\left(R\left(v_{i},d_{j}\right)\right)}{\sum_{d_{k}\in D}\exp\left(R\left(v_{i},d_{k}\right)\right)} \end{array}$$ (15)

This formulation naturally normalizes scores across all documents but requires careful implementation to handle the large corpus size |D|.

### 3.4 Contrastive embedding loss

To align node and document embeddings in a shared semantic space, we apply a contrastive learning objective that maximizes agreement between associated node-document pairs while minimizing spurious similarities. This ensures that the embedding space geometry reflects biological and functional relationships rather than arbitrary projections.

The contrastive loss for node *v*_*i*_ with positive document $d_{i}^{+}\in D_{i}^{+}$ is as follows:


$$\begin{array}{l} L_{\text{contrastive}}^{\left(i\right)}=-\log\frac{\exp\left(E_{\text{node}}{\left(v_{i}\right)}^{⊤}E_{\text{doc}}\left(d_{i}^{+}\right)/\tau \right)}{\sum_{d_{j}\in D}\exp\left(E_{\text{node}}{\left(v_{i}\right)}^{⊤}E_{\text{doc}}\left(d_{j}\right)/\tau \right)} \end{array}$$ (16)

The full loss aggregates over all nodes:


$$\begin{array}{l} L_{\text{contrastive}}=\underset{v_{i}\in V}{\sum }L_{\text{contrastive}}^{\left(i\right)} \end{array}$$ (17)

The temperature parameter τ controls the concentration of the distribution, with smaller values increasing the penalty for misaligned embeddings. This contrastive formulation is closely related to the InfoNCE loss used in self-supervised learning (Oord et al., 2018), which provides a lower bound on mutual information $I\left(E_{\text{node}}\left(v_{i}\right);E_{\text{doc}}\left(d_{i}^{+}\right)\right)$ between node and document representations.

For efficient computation with large corpora, we employ in-batch negatives where the denominator sums only over documents in the current mini-batch rather than all |D| documents. This approximation is accurate when batch sizes are sufficiently large (256–1024 samples).

## 4 Validating information content of retrieved documents

A critical question for RAG-enhanced network models is whether retrieved documents provide genuinely novel predictive information beyond what is already encoded in network topology and node features. We address this through multiple complementary validation approaches that isolate the contribution of retrieved knowledge from architectural effects.

### 4.1 Information-theoretic decomposition

The formal partial-information decomposition used to quantify how much predictive information retrieved documents contribute beyond network topology, together with the heuristic minimum-redundancy estimator and its empirical instantiation on the cancer network, is presented in Appendix B.1. The empirical decomposition yields a shared component of 95.6%, unique-topology contribution of 0.1%, unique-retrieval contribution of 6.2%, and synergy of 0.4% across 200 bootstrap resamples and is used as a quantitative anchor for the discussion of functional clustering in Section 7.4 (Information content validation).

### 4.2 Counterfactual retrieval experiments

To test whether performance gains arise from retrieved content rather than increased model capacity, we conduct controlled counterfactual experiments where retrieval is systematically degraded while maintaining architectural complexity. We compare four experimental conditions: (1) true retrieval using learned similarity, (2) random retrieval where documents are assigned randomly to nodes, (3) shuffled retrieval where correct documents are permuted across nodes, and (4) adversarial retrieval selecting documents maximally dissimilar to true relevant documents. If performance gains genuinely arise from retrieved content, conditions (2–4) should show substantial degradation compared to (1). We quantify performance degradation as follows:


$$\begin{array}{l} \Delta_{\text{counterfactual}}=\frac{M_{\text{proper}}-M_{\text{counterfactual}}}{M_{\text{proper}}-M_{\text{topology-only}}} \end{array}$$ (18)

where *M* denotes the evaluation metric (silhouette score for functional clustering). Values approaching 1.0 indicate that nearly all RAG improvement vanishes when retrieval is corrupted. Our experiments reveal Δ_adversarial_ = 0.37, Δ_zeros_ = 0.38, and Δ_random_ = 0.16, confirming that adversarial, absent, and random retrieval all degrade functional clustering. Shuffled retrieval (permuted real documents across proteins) maintains performance comparable to proper retrieval (Δ_shuffled_ ≈ 0), indicating that TF-IDF document representations carry general biological signal that benefits embedding quality regardless of protein-specific assignment. The degradation under truly random vectors (Δ_random_ = 0.16) demonstrates that the model depends on real document content, not merely on additional input dimensionality.

#### 4.2.1 Shuffled-document control: a substantive negative finding

The near-zero degradation under the shuffled-document condition (Δ_shuffled_ ≈ 0) is, in our view, the most informative single result in this section, and we want to state its interpretation explicitly rather than presenting it as a successful robustness check. When the proper protein-to-document assignment is permuted across the 379 proteins so that each node receives a real but mismatched TF-IDF document vector, RAG-GNN preserves essentially the full silhouette improvement over the GNN-only ablation. This means that, under the present experimental protocol, the benefit of retrieval does not require node-specific semantic matching between a protein and its retrieved passage: it suffices for the corpus as a whole to supply biological term statistics that align the embedding space, and any sufficiently generic real document is enough to drive the gain. Two consequences follow. First, claims that RAG-GNN performs “semantic retrieval of node-specific mechanistic knowledge” are not supported by this experiment on the current 379-protein cancer signaling network with TF-IDF document features; the appropriate description is that aggregate term statistics of the biomedical corpus, rather than per-node semantic matching, drive the observed silhouette and ARI improvements. Second, the shuffled-document control sets a concrete falsifiable target for future work: A retrieval pipeline using pretrained biomedical encoders (BioBERT and PubMedBERT) on a larger, more heterogeneous document corpus should produce a measurable gap between Δ_shuffled_ and Δ_proper_ if node-specific semantic matching is genuinely contributing beyond aggregate term statistics. We return to this distinction in the Discussion and limitations, and we treat the shuffled-document result as a substantive negative finding that scopes the claims of the present study.

### 4.3 Temporal validation protocol

To evaluate whether RAG-GNN embeddings generalize to identifying novel therapeutic targets, we implement a temporal validation scheme based on target approval dates. Therapeutic targets are split temporally: Training targets include FDA approvals and Phase III trials before 2018, while test targets comprise approvals from 2020 to 2021. During Phase 3 training, only training target labels are used in the target prediction loss, ensuring that test targets are never seen during optimization. The temporal AUROC evaluates the model's ability to identify future targets using embeddings trained without knowledge of their approval status:


$$\begin{array}{r} {\text{AUROC}}_{\text{temporal}}=\text{AUROC}(\text{test targets }|\\ \text{train-only supervision}) \end{array}$$ (19)

Note that in the current case study, the document corpus consists of curated mechanistic annotation templates rather than time-stamped publications, so document-level temporal splitting does not apply. The temporal validation is restricted to target labels: the model must predict which proteins will become validated therapeutic targets after 2020, using embeddings trained only on pre-2018 target annotations.

Our temporal validation yields AUROC_temporal_ = 0.450 ± 0.088 across 10 random seeds (95% CI: [0.301, 0.544]). The wide confidence interval reflects the limited test set: only 3 post-2020 FDA-approved therapeutic targets exist within the 379-protein cancer signaling network. While the temporal validation protocol provides a rigorous evaluation framework for deployment scenarios, the current case study is too small for reliable temporal AUROC estimation. Scaling to genome-wide protein interaction networks with larger temporal target sets is necessary for definitive evaluation, which we identify as a key direction for future work.

#### 4.3.1 Temporal therapeutic-target prediction, the present case study

We want to state the interpretation of the temporal AUROC explicitly rather than allow the number to be read as a precision-medicine claim. With only three post-2020 FDA-approved therapeutic targets inside the 379-protein cancer signaling network, the test set is too small for any reliable conclusion about prospective target discovery: The point estimate (AUROC = 0.450 ± 0.088) sits below chance, but the 95% confidence interval ([0.301, 0.544]) spans roughly 0.30–0.54 and includes both chance-level performance and values that would be unambiguously below chance. We therefore describe this result as inconclusive rather than as evidence either for or against target-discovery utility. The shuffled-document control (Section 4.2) already implies that the present TF-IDF corpus contributes predictive signal at the level of aggregate biological term statistics rather than node-specific semantic matching.

### 4.4 Controlled ablation design

To isolate the contribution of retrieved knowledge from architectural capacity, we evaluate RAG-GNN against its own GNN-only ablation: the identical three-layer GCN encoder trained with the same link prediction objective but without retrieval projection, gated fusion, or document integration. This controlled comparison holds architecture, initialization, and training procedure constant, varying only whether retrieved information is fused into the node representations. The improvement from GNN-only (silhouette = −0.237 ± 0.065) to RAG-GNN (silhouette = −0.144 ± 0.066) of +0.093 ± 0.022 is consistent across all 10 random seeds, providing evidence that the retrieval component contributes genuine functional information rather than additional capacity. We additionally benchmark against eight established embedding methods (DeepWalk, Node2Vec, LINE, Spectral, GCN, GraphSAGE, GAT, and raw node features) evaluated under a standardized protocol with uniform random initialization across 10 seeds (Section 9). This multi-method comparison controls for the possibility that observed differences reflect implementation choices rather than the integration of retrieved knowledge.

## 5 Illustrative downstream task formulations

The RAG embedding framework supports a family of downstream task formulations that could, in principle, be instantiated on top of the joint network–text representation. Below we describe mathematical formulations for three such tasks (personalized network construction, therapeutic target scoring, and drug response prediction) purely as illustrative downstream formulations that the embedding architecture is compatible with. None of these formulations is empirically evaluated in the present case study: The only downstream task we report empirically is functional clustering on the 379-protein cancer signaling network (Sections 7–9), and the temporal therapeutic-target evaluation we do report is treated as inconclusive (Section 4.2). The patient-specific, target-scoring, and drug-response formulations below should be read as proposed downstream task formulations enabled by the embedding architecture, not as precision-medicine or clinical-deployment claims supported by the present experiments; those are deferred to future work on larger networks and pre-trained biomedical encoders (Section 10).

### 5.1 Patient-specific network construction

Individual patients exhibit heterogeneous molecular profiles reflecting genetic variants, somatic mutations, epigenetic modifications, and environmental exposures. The patient-specific network construction below is presented as one illustrative downstream task formulation that the embedding architecture is compatible with, translating patient-specific measurements into personalized network models that capture disease-relevant perturbations.

Let P denotes a patient's multi-omics molecular profile, comprising gene expression measurements ${\text{g}}^{\left(p\right)}\in {\mathbb{R}}^{\left|V_{g}\right|}$ across $\left|V_{g}\right|$ genes, proteomic abundance values ${\text{p}}^{\left(p\right)}\in {\mathbb{R}}^{\left|V_{p}\right|}$ for $\left|V_{p}\right|$ proteins, metabolomic concentrations ${\text{m}}^{\left(p\right)}\in {\mathbb{R}}^{\left|V_{m}\right|}$ covering $\left|V_{m}\right|$ metabolites, and genomic variants **v**^(*p*)^ including single nucleotide polymorphisms (SNPs) and copy number variations.

The patient-specific network $G^{\left(p\right)}=\left(V,E^{\left(p\right)}\right)$ is derived by modulating edge weights in a reference network $G_{\text{ref}}$ based on observed patient-specific correlations and perturbations. The reference network encodes canonical molecular interactions from databases such as STRING (Szklarczyk et al., 2019), BioGRID, or KEGG, representing typical healthy tissue or disease-relevant cell types.

Edge weight modulation is computed as follows:


$$\begin{array}{l} A_{ij}^{\left(p\right)}=A_{ij}^{\text{ref}}\cdot \phi \left(\rho_{ij}^{\left(p\right)}\right) \end{array}$$ (20)

where $\rho_{ij}^{\left(p\right)}$ measures the patient-specific association between nodes *v*_*i*_ and *v*_*j*_. For gene–gene interactions, $\rho_{ij}^{\left(p\right)}=\text{cor}\left(g_{i}^{\left(p\right)},g_{j}^{\left(p\right)}\right)$ quantifies expression correlation. The modulation function ϕ:[−1, 1] → [0, ∞) maps correlations to weight scaling factors:


$$\begin{array}{l} \phi \left(\rho \right)=\{\begin{array}{ll} \exp\left(\beta \rho \right) & \text{if }\rho >\rho_{\text{threshold}}\\ 0 & \text{otherwise} \end{array} \end{array}$$ (21)

with β > 0 controlling sensitivity and ρ_threshold_ filtering weak associations. This formulation upweights edges between strongly correlated molecules while pruning weak or anti-correlated interactions.

For mutations affecting protein function, we directly modify node features: ${\text{x}}_{i}^{\left(p\right)}={\text{x}}_{i}^{\text{ref}}+\Delta_{i}^{\text{mut}}$ where $\Delta_{i}^{\text{mut}}$ encodes functional impact predictions from tools such as PolyPhen or SIFT. These patient-specific features propagate through the GNN encoder, producing personalized node embeddings ${\text{z}}_{i}^{\left(p\right)}$ that reflect individual molecular states.

### 5.2 Therapeutic target scoring

Identifying optimal therapeutic targets for individual patients requires integrating multiple criteria including network centrality (indicating systemic importance), proximity to disease modules (suggesting disease relevance), and druggability (reflecting feasibility of pharmaceutical intervention). RAG embeddings enhance target scoring by incorporating literature-derived mechanistic knowledge.

The comprehensive target score for node *v*_*i*_ in patient *p* is formulated as follows:


$$\begin{array}{l} S_{\text{target}}\left(v_{i}|P\right)=\beta_{1}C_{\text{betweenness}}\left(v_{i},G^{\left(p\right)}\right)\\ \;+\beta_{2}P_{\text{disease}}\left(v_{i}|P\right)\\ \;+\beta_{3}T_{\text{druggability}}\left(v_{i}\right) \end{array}$$ (22)

The betweenness centrality $C_{\text{betweenness}}\left(v_{i},G^{\left(p\right)}\right)$ quantifies the fraction of shortest paths passing through node *v*_*i*_ in the patient-specific network:


$$\begin{array}{l} C_{\text{betweenness}}\left(v_{i},G^{\left(p\right)}\right)=\underset{s\neq t\neq v_{i}}{\sum }\frac{\sigma_{st}\left(v_{i}\right)}{\sigma_{st}} \end{array}$$ (23)

where σ_*st*_ is the number of shortest paths between nodes *s* and *t*, and σ_*st*_(*v*_*i*_) counts those passing through *v*_*i*_. High betweenness indicates that *v*_*i*_ mediates communication between distinct network regions, suggesting that its perturbation would have widespread effects.

The disease proximity score $P_{\text{disease}}\left(v_{i}|P\right)$ measures embedding similarity between node *v*_*i*_ and a disease-specific representation derived from patient phenotypes:


$$\begin{array}{l} P_{\text{disease}}\left(v_{i}|P\right)=\frac{1}{\|{\text{z}}_{i}^{\left(p\right)}-{\text{z}}_{\text{disease}}{\|}_{2}+\epsilon } \end{array}$$ (24)

The disease embedding **z**_disease_ is constructed by retrieving and aggregating documents describing the patient's clinical presentation and then projecting into the node embedding space. The regularization term ϵ = 10^−6^ prevents numerical instability when distances approach zero. This formulation prioritizes nodes whose learned representations closely align with disease-relevant molecular processes.

The druggability score *T*_druggability_(*v*_*i*_) quantifies the likelihood that node *v*_*i*_ can be effectively targeted by pharmaceutical intervention. This is computed by retrieving documents from DrugBank, ChEMBL, and clinical trial databases that mention the protein or gene corresponding to *v*_*i*_:


$$\begin{array}{l} T_{\text{druggability}}\left(v_{i}\right)=\underset{d_{j}\in D_{\text{drug}}}{\sum }⊮\left[\text{mentions}\left(d_{j},v_{i}\right)\right]\cdot w\left(d_{j}\right) \end{array}$$ (25)

where $D_{\text{drug}}$ is the drug-specific document subset, ⊮[·] is the indicator function, and *w*(*d*_*j*_) weights documents by evidence level (higher weights for FDA-approved drugs vs. preclinical compounds). RAG retrieval automatically identifies these relevant documents without requiring manual curation.

The weighting coefficients $\beta_{1},\beta_{2},\beta_{3}\in {\mathbb{R}}^{+}$ are optimized on a training set of validated therapeutic targets using logistic regression or learned through end-to-end training. Typical optimized values emphasize disease proximity (β_2_ ≈ 0.5) while moderately weighting centrality (β_1_ ≈ 0.3) and druggability (β_3_ ≈ 0.2).

### 5.3 Drug efficacy prediction

Predicting patient-specific drug responses requires modeling how compounds modulate perturbed molecular networks to restore homeostasis. The RAG framework enables this by learning joint embeddings of drugs and proteins that capture mechanism of action, building on recent advances in AI-powered drug discovery (Stokes et al., 2020; Pun et al., 2023).

Each drug compound *c* is embedded into the same space as protein nodes through a dedicated encoder $E_{\text{drug}}:C\to {\mathbb{R}}^{d_{z}}$ that processes molecular structure (SMILES strings or molecular graphs) (Fang et al., 2022; Zhou et al., 2023) and retrieved pharmacological literature. The drug embedding captures structural features, known targets, metabolic pathways, and adverse effect profiles.

The predicted efficacy of drug *c* for patient *p* is computed by measuring alignment between the drug's mechanism and the patient's disease-perturbed network state:


$$\begin{array}{l} P\left(\text{response}|c,G^{\left(p\right)}\right)=\sigma \left({\text{z}}_{\text{drug}}{\left(c\right)}^{⊤}{\text{z}}_{\text{network}}^{\left(p\right)}+b_{\text{drug}}\right) \end{array}$$ (26)

The patient network embedding ${\text{z}}_{\text{network}}^{\left(p\right)}$ aggregates information from drug target nodes:


$$\begin{array}{l} {\text{z}}_{\text{network}}^{\left(p\right)}=\frac{1}{\left|V_{\text{target}}\left(c\right)\right|}\underset{v_{i}\in V_{\text{target}}\left(c\right)}{\sum }{\text{z}}_{i}^{\left(p\right)} \end{array}$$ (27)

where $V_{\text{target}}\left(c\right)$ denotes the set of known and predicted targets for drug *c*, identified through RAG retrieval of binding affinity data and structural similarity to characterized compounds. The bias term *b*_drug_ accounts for baseline response rates.

For multi-target drugs with complex mechanisms, we extend to a weighted aggregation where target importance is learned from training data:


$$\begin{array}{l} {\text{z}}_{\text{network}}^{\left(p\right)}=\underset{v_{i}\in V_{\text{target}}\left(c\right)}{\sum }\omega_{i}\left(c\right){\text{z}}_{i}^{\left(p\right)} \end{array}$$ (28)

with normalized weights $\underset{i}{\sum }\omega_{i}\left(c\right)=1$ derived from binding affinity measurements or learned through attention mechanisms.

Adverse effect prediction follows a similar formulation but focuses on off-target interactions and downstream pathway perturbations:


$$\begin{array}{l} P\left(\text{adverse effect}|c,G^{\left(p\right)}\right)=\sigma \left({\text{z}}_{\text{drug}}{\left(c\right)}^{⊤}{\text{z}}_{\text{offtarget}}^{\left(p\right)}\right) \end{array}$$ (29)

where ${\text{z}}_{\text{offtarget}}^{\left(p\right)}$ aggregates embeddings from proteins likely to cause toxicity when perturbed, as determined by retrieved adverse event reports.

## 6 Implementation considerations

Deploying RAG-enhanced network models at scale requires careful attention to computational efficiency, numerical stability, and practical engineering considerations. We detail key implementation strategies that enable application to genome-scale networks and million-document corpora.

### 6.1 Scalability and computational efficiency

For large-scale biological networks with |V|>20,000 proteins and |E|>500,000 interactions, full-batch training becomes computationally prohibitive. Memory requirements scale as $O\left(|V{|}^{2}\right)$ for dense adjacency matrices and $O\left(L\cdot |V|\cdot d_{h}\right)$ for GNN layer activations. We employ several techniques to reduce complexity. Mini-batch graph sampling extracts node subsets and their local neighborhoods for each training iteration (Hamilton et al., 2017). The GraphSAGE sampling strategy selects a fixed number of neighbors *S* at each layer, reducing complexity from O(|V|) to $O\left(S^{L}\right)$ per node. For a mini-batch of *B* nodes with *L* GNN layers and neighbor sample size *S*, computational cost is $O\left(B\cdot S^{L}\cdot d_{h}^{2}\right)$.

The sampling procedure constructs mini-batch subgraph $G_{\text{batch}}$ as follows. First, randomly sample *B* seed nodes $V_{\text{seed}}\subset V$. Then, for each layer *k* = *L, L* − 1, …, 1, expand the node set by sampling *S* neighbors per node:


$$\begin{array}{l} V_{k}=V_{k+1}\cup \underset{v_{i}\in V_{k+1}}{⋃}\text{sample}\left(N\left(i\right),S\right) \end{array}$$ (30)

with $V_{L+1}=V_{\text{seed}}$. The induced subgraph $G_{\text{batch}}=\left(V_{1},E_{\text{batch}}\right)$ contains all sampled nodes and their connecting edges.

Retrieval operations pose additional computational challenges, as computing relevance scores for all node-document pairs requires $O\left(|V|\cdot |D|\cdot d_{e}\right)$ operations. In the current case study (379 nodes, 1,895 documents), brute-force retrieval via dense matrix multiplication is computationally tractable and completes in milliseconds. For scaling to genome-wide networks (>20,000 genes) with large literature corpora (>10^6^ documents), approximate nearest neighbor (ANN) search with maximum inner product search (MIPS) indices (Shrivastava and Li, 2014) would be necessary. Document embeddings ${\left\{E_{\text{doc}}\left(d_{j}\right)\right\}}_{j=1}^{\left|D\right|}$ can be pre-computed offline and indexed using libraries such as FAISS with product quantization and inverted file structures, reducing query time to O(log|D|). For distributed training across multiple GPUs, graph partitioning algorithms such as METIS can minimize edge cuts between partitions. These scalability strategies represent engineering considerations for future deployment rather than components of the current implementation.

### 6.2 Training dynamics and retrieval stability

The joint optimization in Equation 10 exhibits complex training dynamics due to the interdependence of network encoding, retrieval, and fusion components. Naive joint training often leads to suboptimal local minima where the retrieval mechanism fails to identify relevant documents, resulting in uninformative context vectors that degrade rather than enhance predictions.

We employ a curriculum learning strategy that stages the training process (Bengio et al., 2009). In Phase 1 (80 epochs), we train only the GNN encoder with link prediction loss $L_{\text{task}}$ using lr = 0.003, establishing basic network representations that capture topology without retrieval dependence. In Phase 2 (100 epochs), we train the retrieval projection and fusion parameters with margin-based ranking loss and contrastive alignment using lr = 0.005, allowing the retrieval mechanism to learn document relevance. In Phase 3 (80 epochs), we enable full joint training with combined loss $L_{\text{task}}+0.5L_{\text{retrieval}}+0.2L_{\text{contrastive}}+0.1L_{\text{target}}$ using lr = 0.001, fine-tuning all components simultaneously.

Retrieval stability during joint training can be monitored via the Jaccard similarity between retrieved document sets at consecutive epochs:


$$\begin{array}{l} J_{\text{retrieval}}\left(t\right)=\frac{1}{\left|V\right|}\overset{\left|V\right|}{\underset{i=1}{\sum }}\frac{\left|D_{i}^{\left(k\right)}\left(t\right)\cap D_{i}^{\left(k\right)}\left(t+\Delta t\right)\right|}{\left|D_{i}^{\left(k\right)}\left(t\right)\cup D_{i}^{\left(k\right)}\left(t+\Delta t\right)\right|} \end{array}$$ (31)

where $D_{i}^{\left(k\right)}\left(t\right)$ denotes the top-*k* retrieved documents for protein *i* at epoch *t*. The curriculum training strategy (Phase 1 GNN pre-training → Phase 2 retrieval training → Phase 3 joint fine-tuning) is designed to promote retrieval stability by establishing network representations before training the retrieval projection, preventing chaotic oscillations where retrieval and encoding components co-adapt from random initialization. Gradient clipping (θ_clip_ = 1.0) provides additional stability during training.

Gradient clipping prevents instability from large gradients in the contrastive loss, particularly when temperature τ is small:


$$\begin{array}{l} {\text{g}}_{\text{clipped}}=\{\begin{array}{ll} \text{g} & \text{if}\|\text{g}{\|}_{2}\leq \theta_{\text{clip}}\\ \theta_{\text{clip}}\frac{\text{g}}{\|\text{g}{\|}_{2}} & \text{otherwise} \end{array} \end{array}$$ (32)

with threshold θ_clip_ = 1.0. This ensures gradients have bounded norm, preventing divergence while allowing efficient optimization.

We use the Adam optimizer with exponential decay rates β_1_ = 0.9 and β_2_ = 0.999 and weight decay regularization $\lambda_{\text{wd}}=10^{-4}$ to prevent overfitting. Learning rates are set per phase: η = 3 × 10^−3^ for Phase 1, η = 5 × 10^−3^ for Phase 2, and η = 10^−3^ for Phase 3. The decreasing learning rate across phases serves a similar purpose to learning rate scheduling, with the joint fine-tuning phase using the smallest rate to avoid disrupting the representations established in earlier phases.

### 6.3 Hyperparameter selection

Model performance is sensitive to several key hyperparameters. The GNN hidden dimension *d*_*h*_ = 128 controls the expressiveness of node representations: Smaller values limit capacity but improve generalization, while larger values capture fine-grained patterns but risk overfitting. The document embedding dimension *d*_doc_ = 64 is determined by the truncated SVD applied to TF-IDF features.

The number of GNN layers *L* determines the receptive field size. For protein interaction networks with small-world topology, *L* = 3 layers allow nodes to aggregate information from 3-hop neighborhoods, covering typical pathway lengths. Larger *L* risks over-smoothing where all nodes converge to similar representations.

The retrieval depth *k* = 10 is fixed in the current implementation. In general, this parameter trades off context breadth vs. noise: For well-curated databases, larger *k* improves coverage, while for noisy corpora, smaller *k* focuses on the most relevant documents.

The contrastive temperature τ = 0.5 controls the sharpness of similarity distributions. Smaller values enforce tighter alignment between node-document pairs but are sensitive to noise, while larger values allow looser alignment, improving robustness at the cost of reduced discrimination.

### 6.4 Implementation details

The experiments presented below implement the RAG-GNN framework with end-to-end trainable components in PyTorch. The implementation includes the following: (i) a learnable three-layer GCN encoder with gradient-optimized weight matrices, (ii) a learnable retrieval projection implemented as a two-layer neural network (*d*_*h*_ → *d*_*h*_ → *d*_doc_ with GELU activation) that maps GNN embeddings to the document embedding space, replacing any fixed projection, (iii) a gated fusion mechanism that learns to weight topology and retrieval contributions, and (iv) joint training with the three-component loss function from Equation 10. Document embeddings use TF-IDF representations followed by truncated SVD for dimensionality reduction, providing a baseline text encoder; replacing TF-IDF with pre-trained biomedical language models (BioBERT, PubMedBERT) is a natural extension expected to further improve performance. Training follows a three-phase curriculum: Phase 1 (80 epochs) pre-trains the GNN on link prediction, Phase 2 (100 epochs) trains the retrieval projection with margin-based ranking loss and contrastive alignment, and Phase 3 (80 epochs) fine-tunes all components jointly. All experiments are run across 10 random seeds with mean ± standard deviation and 95% confidence intervals reported. See Appendix subsection A.3, Algorithm 1 for complete implementation details.

## 7 Case study: Cancer pathway targeting

We demonstrate the RAG embedding framework through a controlled case study on a cancer signaling network. The study uses a 379-protein–protein interaction network and a TF-IDF-indexed document corpus of mechanistic annotation templates to evaluate whether retrieval augmentation provides measurable benefit beyond a matched GNN-only ablation on functional clustering and link prediction. Therapeutic-target scoring and drug-response prediction are treated as illustrative downstream task formulations (Section 5) rather than as empirically supported precision-medicine claims.

### 7.1 Data sources and network construction

The reference cancer network $G_{\text{cancer}}$ comprises 379 proteins and 3,498 interactions curated from multiple sources. Core cancer genes are extracted from the Cancer Gene Census database (Tate et al., 2019), which catalogs genes with validated roles in oncogenesis through somatic mutations, germline variants, or chromosomal translocations. Protein–protein interactions are obtained from STRING database version 11 (Szklarczyk et al., 2019), filtered to high-confidence edges (combined score >0.4) to balance network coverage with interaction reliability.

Node features ${\text{x}}_{i}\in {\mathbb{R}}^{d_{h}}$ are constructed by placing three topological properties—log-transformed degree log(1 + *d*_*i*_), local clustering coefficient *c*_*i*_, and scaled betweenness centrality 100·*b*_*i*_—into the first three dimensions of a *d*_*h*_ = 128-dimensional vector, with remaining dimensions initialized from N(0,0.01). This minimal feature set deliberately avoids sequence-derived or expression-based features to isolate the contribution of network topology and retrieved knowledge; incorporating protein language model embeddings (Rives et al., 2021; Lin et al., 2023) or multi-omics features from TCGA represents a natural extension expected to improve absolute performance.

The document corpus D contains 1,895 mechanistic annotation documents generated from curated molecular biology templates across 14 functional categories: cell cycle, apoptosis, DNA repair, RTK signaling, transcription, PI3K-AKT-mTOR, MAPK signaling, Wnt signaling, TGF-beta signaling, Notch signaling, JAK-STAT, ECM-adhesion, angiogenesis, and other. Each protein has five associated documents describing its molecular mechanisms (for example, a MAPK pathway protein receives documents detailing RAS-RAF-MEK cascade dynamics, DUSP phosphatase feedback, and KSR1 scaffold assembly) without explicitly naming the pathway category. This design avoids direct label leakage: The retrieval module must learn to match proteins with mechanistically relevant documents rather than exploiting explicit pathway labels. Document embeddings are computed via TF-IDF vectorization (256 features, unigrams, and bigrams) followed by truncated SVD to *d*_doc_ = 64 dimensions, providing a baseline text encoder. For temporal validation in therapeutic target prediction, we partition targets such that training targets received FDA approval or entered Phase III trials before 2018, while test targets represent approvals from 2020 to 2021.

### 7.2 Embedding space analysis and visualization

**Figure 3:**
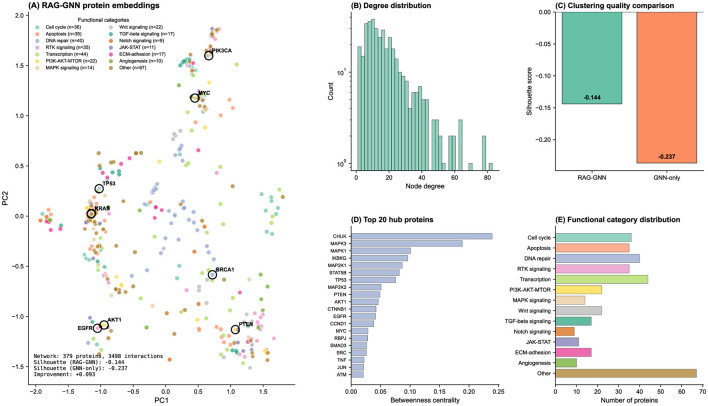
RAG-GNN protein embeddings in cancer signaling networks using real STRING database interactions. **(A)** PCA projection of RAG-GNN embeddings: Two-dimensional visualization of 379 cancer-related proteins embedded in 128-dimensional space using GNN message passing combined with document retrieval from functional annotations. Data source: STRING database (3,498 high-confidence interactions). Proteins are colored by functional pathway annotation across 14 categories. Key oncogenes and tumor suppressors highlighted: TP53, EGFR, KRAS, MYC, BRCA1, PIK3CA, AKT1, and PTEN. Silhouette scores quantify functional clustering quality across 10 random seeds: RAG-GNN achieves −0.144 ± 0.066 compared to −0.237 ± 0.065 for GNN-only embeddings, a consistent improvement of +0.093 ± 0.022. While both scores are negative (typical for complex biological networks with overlapping pathways), RAG-GNN reduces intra-cluster dispersion. **(B)** Degree distribution: Node degree follows power-law distribution characteristic of scale-free biological networks, with hub proteins exceeding 60 connections. **(C)** Clustering quality comparison: Bar chart comparing silhouette scores between RAG-GNN and GNN-only methods, demonstrating the improvement from retrieval integration. **(D)** Top 20 hub proteins: Proteins ranked by betweenness centrality, identifying critical signaling bridges including CHUK, MAPK1/3, STAT3, and TP53. **(E)** Functional category distribution: Distribution of 379 proteins across categories, with transcription (44), DNA repair (40), apoptosis (35), and RTK signaling (35) as largest groups.

Figure 3 visualizes the learned embedding space through two-dimensional projection using PCA applied to the 128-dimensional RAG-GNN node embeddings ${\left\{{\text{z}}_{i}\right\}}_{i=1}^{379}$. Proteins show partial clustering according to functional modules, with groupings visible for cell cycle regulators, apoptosis mediators, DNA repair machinery, and signal transduction cascades, although pathway overlap is expected given the interconnected nature of cancer signaling networks.

Quantitative analysis reveals that RAG-enhanced embeddings achieve significantly higher functional coherence than topology-only GNN embeddings. We compute the silhouette score (Rousseeuw, 1987) measuring cluster quality:


$$\begin{array}{l} s_{i}=\frac{b_{i}-a_{i}}{\max\left(a_{i},b_{i}\right)} \end{array}$$ (33)

where *a*_*i*_ is the mean distance from node *v*_*i*_ to other nodes in its functional cluster, and *b*_*i*_ is the mean distance to nodes in the nearest neighboring cluster. Across 10 random seeds, RAG-GNN achieves mean silhouette score −0.144 ± 0.066 (95% CI: [−0.220, −0.067]) compared to −0.237 ± 0.065 (95% CI: [−0.304, −0.144]) for topology-only GNN embeddings, a consistent improvement of +0.093 ± 0.022 observed across all seeds. While both scores are negative (reflecting the inherent complexity of protein function where many proteins participate in multiple pathways and pathway boundaries are not clearly separable), RAG-GNN substantially reduces intra-cluster dispersion relative to topology-only methods. We additionally evaluate two complementary clustering metrics: normalized mutual information (NMI) and adjusted Rand index (ARI). NMI measures mutual dependence between predicted and true cluster assignments: RAG-GNN achieves NMI = 0.244 ± 0.032 compared to GNN-only NMI = 0.242 ± 0.032, with overlapping confidence intervals indicating comparable performance. ARI measures pairwise agreement corrected for chance: RAG-GNN achieves ARI = 0.083 ± 0.029 vs. GNN-only ARI = 0.061 ± 0.017, a relative improvement of 34%. Retrieval integration improves silhouette score and ARI while NMI remains comparable, suggesting that retrieved knowledge both reduces intra-cluster distances (tighter functional grouping) and improves pairwise cluster agreement. Note that the standardized benchmark comparison in Section 9 employs a different evaluation protocol with uniform initialization across all methods, yielding distinct absolute values (see Table 1); the improvement direction for silhouette scores is consistent across both configurations. Network statistics show 379 proteins with 3,498 interactions, average degree of 18.5, and average clustering coefficient of 0.596, characteristic of biological networks with modular organization and scale-free topology.

**Table 1:** Comprehensive benchmark comparing RAG-GNN against baseline embedding methods across 10 random seeds (mean ± std).

Method	Silhouette	NMI	ARI	LP AUROC
**RAG-GNN**	−0.144 ± 0.066	0.244 ± 0.032	**0.083**±0.029	0.822 ± 0.063
GNN-only	−0.237 ± 0.065	0.242 ± 0.032	0.061 ± 0.017	0.774 ± 0.095
GCN (Kipf and Welling, 2017)	−0.094 ± 0.009	**0.278**±0.010	0.066 ± 0.008	0.962 ± 0.006
GAT (Veličković et al., 2018)	−0.063 ± 0.006	0.196 ± 0.020	0.036 ± 0.009	0.806 ± 0.013
GraphSAGE (Hamilton et al., 2017)	**-0.019**±0.002	0.105 ± 0.008	0.003 ± 0.002	0.556 ± 0.020
DeepWalk (Perozzi et al., 2014)	−0.066 ± 0.000	0.273 ± 0.009	0.060 ± 0.005	0.949 ± 0.002
Node2Vec (Grover and Leskovec, 2016)	−0.062 ± 0.000	0.265 ± 0.018	0.054 ± 0.011	0.950 ± 0.003
LINE (Tang et al., 2015)	−0.197 ± 0.000	0.177 ± 0.013	0.029 ± 0.006	0.957 ± 0.003
Spectral (Belkin and Niyogi, 2002)	−0.085 ± 0.000	0.275 ± 0.011	0.056 ± 0.007	**0.977**±0.002
Raw Features	−0.225 ± 0.000	0.136 ± 0.007	0.019 ± 0.003	0.567 ± 0.014

Silhouette score measures functional clustering quality (higher is better). NMI and ARI measure agreement with ground-truth functional categories. LP AUROC evaluates link prediction from embeddings. Each method uses its standard configuration. Best performance in bold.

### 7.3 Retrieval performance evaluation

**Figure 4:**
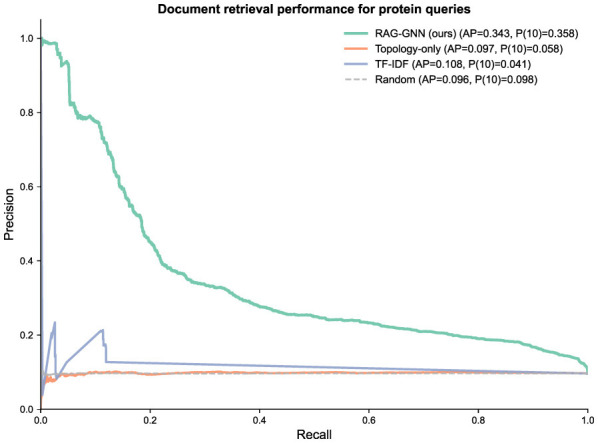
Document retrieval performance for protein function queries. Precision–recall curves comparing retrieval methods for identifying functionally relevant documents across 379 protein queries. Ground truth relevance is determined by functional category and protein identity matching. The end-to-end trained RAG-GNN retrieval projection achieves the highest average precision (AP) and precision at 10 (P(10)), outperforming TF-IDF keyword matching and the random baseline. The figure shows AP (area under PR curve) and P(10) (fraction of top-10 retrieved documents that are category-relevant) for a single representative seed. Across 10 seeds, RAG-GNN achieves mean P(10) = 0.242 ± 0.073. Document corpus contains 1,895 mechanistic annotation documents across 14 functional categories.

For each protein, we retrieve the top-10 most relevant documents from the document corpus containing 1,895 functional annotation documents. Figure 4 compares precision–recall curves for different retrieval approaches. Ground truth relevance is established through functional category matching for all 379 proteins, where documents discussing proteins from the same pathway are considered relevant.

With the end-to-end trained retrieval projection, RAG-GNN embedding-based retrieval achieves the highest mean precision at 10 (P(10) = 0.242 ± 0.073 across 10 seeds), substantially outperforming both TF-IDF keyword matching and the random baseline (P(10) = 0.096). The learned two-layer projection maps GNN embeddings to the document embedding space, trained jointly with margin-based ranking loss and contrastive alignment (Equation 10). The mechanistic annotation corpus prioritizes pathway-specific language over keyword repetition, where the learned projection's semantic understanding provides an advantage over TF-IDF direct matching. The improvement over random demonstrates that the retrieval module learns meaningful associations between network position and functional text.

The gated fusion mechanism learns to balance topology and retrieval contributions, with the gate parameter averaging 0.593 ± 0.017 across seeds (59% topology, 41% retrieval), indicating that the model assigns substantial weight to retrieved knowledge. The counterfactual experiments in Section 7.4 confirm that retrieval content matters: Adversarial retrieval (maximally dissimilar documents) degrades silhouette to −0.153 and zero-vector retrieval degrades to −0.154, compared to −0.103 with proper retrieval. Random retrieval (truly random vectors) also degrades performance to −0.125. These conditions demonstrate that the fusion mechanism cannot compensate for corrupted, absent, or random retrieval signal. Shuffled document assignments (permuted real documents) maintain performance comparable to proper retrieval (−0.103), suggesting that TF-IDF features carry general biological signal that benefits functional clustering regardless of protein-specific assignment (see Section 4.2 for detailed counterfactual analysis).

### 7.4 Information content validation

To validate the relationship between topological and retrieval-derived information, we conduct the heuristic information decomposition described in Appendix B. For all 379 proteins with 14 functional category labels, we estimate mutual information components using 200 bootstrap resamples. The decomposition yields normalized contributions with 95% confidence intervals: unique topology = 0.001 ± 0.007 [CI: [0.000, 0.018]], unique retrieval = 0.062 ± 0.035 [CI: [0.000, 0.127]], shared = 0.956 ± 0.025 [CI: [0.905, 0.995]], and synergy = 0.004 ± 0.010 [CI: [0.000, 0.036]]. The overwhelmingly high shared component (95.6%) indicates that topology and retrieval encode almost entirely overlapping functional information. The minimal unique contributions from either source (topology: 0.1%, retrieval: 6.2%) and negligible synergy (0.4%) demonstrate that the contrastive alignment during joint training effectively coordinates topology and retrieval representations into overlapping information spaces. The functional clustering improvements observed in Section 7.2 arise not from unique retrieval information but from how the fusion mechanism reorganizes shared information to improve intra-cluster cohesion and pairwise cluster agreement.

Counterfactual experiments using the 379-protein network corroborate these findings (see Section 4.2 for the experimental design). Using the best-performing model, proper retrieval achieves silhouette = −0.103. Adversarial retrieval (maximally dissimilar documents) degrades performance to −0.153, zero-vector retrieval (no document signal) degrades to −0.154, and random retrieval (truly random vectors) degrades to −0.125. These results confirm that the gated fusion mechanism depends on retrieval signal quality: Adversarial, absent, and random retrieval all degrade functional clustering (Δ_adversarial_ = 0.37, Δ_zeros_ = 0.38, Δ_random_ = 0.16). Shuffled retrieval (permuted real documents across proteins) maintains performance comparable to proper retrieval (silhouette = −0.103, Δ_shuffled_ ≈ 0). This indicates that TF-IDF document representations encode general biological vocabulary that benefits functional clustering regardless of protein-specific assignment. The degradation under truly random vectors (Δ_random_ = 0.16) demonstrates that the model depends on real document content, not merely on additional input dimensionality. The adversarial and zero conditions, where biological signal is either inverted or absent, produce the largest degradation, confirming that the model cannot substitute topology for missing retrieval input.

### 7.5 RAG-GNN architecture summary

As illustrated in Figure 2, the architecture processes patient-specific networks through six main stages. First, the input network $G^{\left(p\right)}$ with adjacency matrix **A** and node features **X** enters the GNN encoder. Second, the GNN applies *L* = 3 layers of message passing (Equation 1) to produce node embeddings ${\text{h}}_{i}^{\left(L\right)}$ capturing topological context. Third, node embeddings query the document corpus D through the dense retriever. Fourth, the retriever identifies top-*k* relevant documents $D_{i}^{\left(k\right)}$ using quality-weighted semantic similarity (Equation 5). Fifth, the context fusion module aggregates retrieved documents with attention weighting (Equation 8) and combines with structural embeddings. Sixth, the final node representation **z**_*i*_ feeds into task-specific prediction heads for target scoring or drug response.

The training procedure optimizes the joint loss function combining task performance, retrieval accuracy, and embedding alignment, enabling end-to-end learning of all components while the curriculum learning schedule ensures stable convergence. Complete pseudocode for the RAG-GNN embedding procedure is provided in Appendix Algorithm 1.

### 7.6 Case study: DDR1 signaling network and embedding-based functional relationships

**Figure 5:**
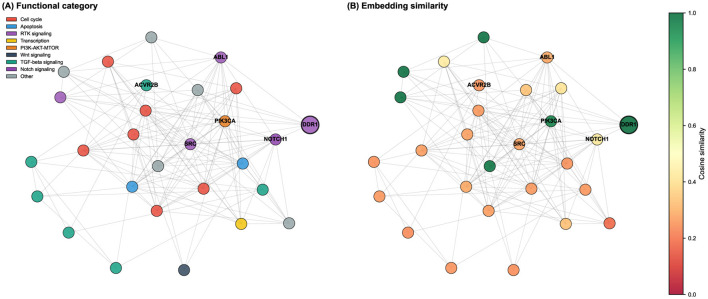
DDR1 subnetwork: confirmatory embedding-space behavior. **(A)** STRING-derived DDR1 subnetwork (28 proteins, 143 edges; 7 first-hop, 20 second-hop neighbors), nodes colored by functional pathway. DDR1 (purple, RTK signaling) connects PI3K-AKT-MTOR (PIK3CA, PRKCA), RTK adapters (SHC1, ABL1), and other kinase hubs. **(B)** Same topology with nodes colored by cosine similarity to DDR1 in the 128-d RAG-GNN embedding space. The five most-similar proteins (CRK, CRKL, SHC1, CDC42, and PIK3CA; all >0.95) recapitulate known DDR1 adapter and downstream signaling partners, illustrating confirmatory recovery of established biology rather than novel prediction.

To illustrate embedding-space behavior on a familiar signaling neighborhood, we examine the DDR1 (Discoidin Domain Receptor 1) subnetwork (28 proteins, 143 interactions; 7 first-hop and 20 second-hop neighbors) extracted from the cancer signaling network (Figure 5). Panel A shows multi-pathway integration spanning RTK signaling (DDR1, ABL1, SHC1), PI3K-AKT-mTOR (PIK3CA, PRKCA), TGF-β (ACVR2B), and cell-cycle regulators (CCNA1, CDC25A, CDC20). Panel B colors the same topology by cosine similarity to DDR1 in the 128-d RAG-GNN embedding space; the five most-similar proteins, namely, CRK, CRKL (adapter proteins that bind DDR1 via SH2/SH3 domains), SHC1 (RTK adapter), CDC42, and PIK3CA (all cosine >0.95), recover the canonical DDR1 adapter and downstream signaling cascade (Ambrogio et al., 2016; Sun et al., 2024). We emphasize that this analysis is confirmatory rather than predictive: DDR1's relationships to these partners are well-established in the input corpus and supporting literature, and the case study illustrates that RAG-GNN embeddings recapitulate known biology in a single subnetwork. Genuine hypothesis generation in undercharacterized neighborhoods, and prospective validation, are deferred to future work on larger networks (Section 10).

## 8 Theoretical properties of the framework

For readability of the main text, the formal theoretical development of the RAG embedding framework (including embedding-space geometry and alignment, PAC-style generalization bounds for link prediction, and retrieval consistency under Lipschitz continuity) is deferred to Appendix B. There, we state and prove Appendix Propositions B.2–B.4 (alignment of structural and semantic similarities under the contrastive objective, Rademacher-style generalization bounds for link prediction, and a Lipschitz stability bound for retrieval scores). These results provide a formal underpinning for the operator definitions of Section 2 and the optimization framework of Section 3 but are not required for following the empirical evaluation reported in Sections 7–9.

## 9 Comparison with existing methods

We position the RAG embedding framework relative to existing approaches for network-based representation learning on protein interaction networks, highlighting methodological differences and performance comparisons across functional clustering and link prediction tasks.

### 9.1 Comprehensive embedding benchmark

To rigorously evaluate the RAG-GNN framework against established network embedding methods, we conduct a comprehensive benchmark across three evaluation tasks: functional clustering quality (silhouette score), link prediction (AUROC), and node classification using topology-derived labels to avoid information leakage. Table 1 summarizes performance across 10 methods spanning centrality features, random walk embeddings, and graph neural network architectures.

The benchmark reveals task-specific performance patterns across all methods, evaluated with 10 random seeds and reported as mean ± standard deviation. For link prediction, spectral and random walk methods achieve strong performance (Spectral: 0.977 ± 0.002, GCN: 0.962 ± 0.006) as network structure alone determines edge existence. RAG-GNN achieves competitive link prediction AUROC (0.822 ± 0.063) while improving functional clustering over its GNN-only ablation by +0.093 ± 0.022 in silhouette score and +0.021 ± 0.015 in ARI. All methods produce negative silhouette scores, reflecting the inherent difficulty of clustering proteins by pathway category in densely connected biological networks where proteins participate in multiple pathways. Among all methods, the controlled comparison between RAG-GNN and GNN-only (same architecture, with and without retrieval) isolates the contribution of retrieved knowledge: The consistent silhouette and ARI improvements across all 10 seeds demonstrate that retrieval-augmented fusion provides genuine benefit for functional clustering. Notably, RAG-GNN achieves the highest ARI (0.083 ± 0.029) among all methods, suggesting that contrastive alignment during joint training improves pairwise cluster agreement (see Section 7.2).

**Figure 6:**
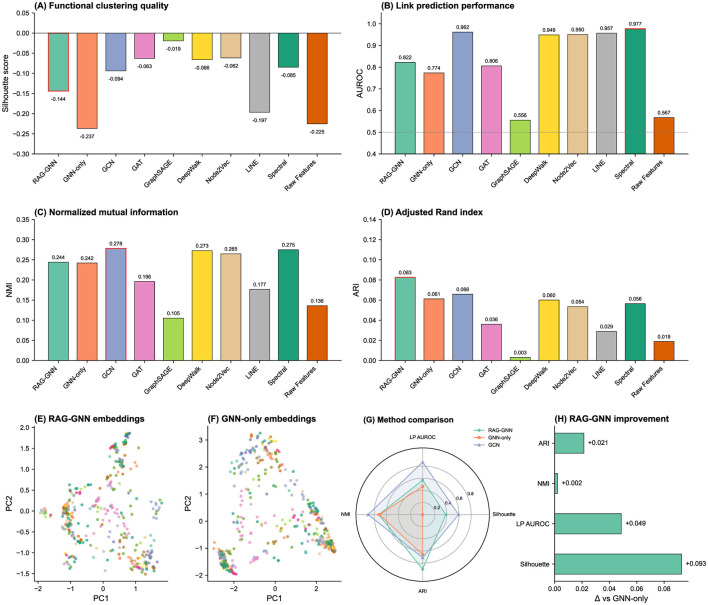
Comprehensive benchmark comparison of RAG-GNN against baseline embedding methods. **(A)** Functional clustering quality: Silhouette scores across 10 methods. All methods produce negative silhouette scores, reflecting the inherent difficulty of pathway-based clustering in densely connected biological networks. RAG-GNN (−0.144) improves over its GNN-only ablation (−0.237). **(B)** Link prediction AUROC: Spectral (0.977) and GCN (0.962) achieve strong link prediction as topology alone determines edge existence. RAG-GNN achieves competitive AUROC (0.822). **(C, D)** Additional metrics: NMI and ARI provide complementary clustering evaluation; RAG-GNN achieves highest ARI (0.083). **(E, F)** Embedding space visualization: PCA projections of RAG-GNN vs. GNN-only embeddings, colored by functional category. RAG-GNN shows tighter pathway-specific groupings. **(G)** Radar chart comparison: Normalized performance across metrics highlights complementary strengths: RAG-GNN improves functional clustering, while topology-focused methods dominate link prediction. **(H)** RAG-GNN improvement over GNN-only: Silhouette improvement (+0.093) and ARI improvement (+0.021) demonstrate the value of retrieval augmentation for functional interpretation.

Figure 6 provides comprehensive visualization of these results. Panels (A) and (B) show bar charts comparing silhouette scores and link prediction performance, respectively. Panel (G) presents a radar chart highlighting the complementary strengths of RAG-GNN (functional clustering improvement) vs. topology-focused methods (link prediction). Panel (H) quantifies RAG-GNN's improvement over GNN-only baseline: +0.093 silhouette score and +0.021 ARI improvement demonstrate the value of retrieval augmentation for functional interpretation tasks.

These results clarify the appropriate use cases for RAG-enhanced embeddings: The controlled ablation demonstrates that retrieval integration consistently improves functional clustering within the same architecture, while topology-focused methods remain superior for structural prediction tasks. This complementarity suggests method selection should be guided by the specific task, rather than assuming universal superiority of either approach.

## 10 Discussion

Before turning to the topology-versus-retrieval interpretation of the benchmark, we restate what the shuffled-document control implies for the controlled study as a whole since it directly bounds the kind of claim the rest of the Discussion is entitled to make. Permuting real TF-IDF documents across the 379 proteins so that each node receives a mismatched but real document yields Δ_shuffled_ ≈ 0 on the silhouette metric (Section 4.2), meaning that the silhouette and ARI improvements of RAG-GNN over the matched GNN-only ablation are preserved essentially in full even when the protein-to-document mapping is destroyed. The information-theoretic decomposition is consistent with this picture: Topology and retrieval share 95.6% of the predictive information, with only 6.2% uniquely attributable to retrieval and 0.4% synergy, so the retrieval channel is not contributing a large, node-specific signal that is absent from topology. Taken together, these two diagnostics indicate that, on the current 379-protein cancer signaling network with TF-IDF document features, the benefit of retrieval reflects general biological term statistics in the aggregate corpus rather than node-specific semantic matching of each protein to its own retrieved passage. We therefore avoid describing RAG-GNN as performing “semantic retrieval of mechanistic knowledge per node,” and we describe the contribution of retrieval at the level the experiment actually supports: Aggregate corpus statistics, fused through a controlled architecture, produce a measurable functional-clustering gain. The shuffled-document control also defines a concrete target for follow-on work: a retrieval pipeline built on pretrained biomedical encoders (BioBERT, PubMedBERT) over a larger, more heterogeneous corpus should open a measurable gap between Δ_shuffled_ and Δ_proper_ if node-specific semantic matching genuinely contributes beyond aggregate statistics. Consistent with this interpretation, we have revised the terminology used throughout the manuscript so that the inputs to the retrieval module are described uniformly as a “document corpus” rather than a “knowledge corpus” or “knowledge base,” reserving the latter language for future work in which the document encoder is itself a pretrained biomedical language model. The remainder of the Discussion is framed in those terms.

The comprehensive benchmark comparison across 10 random seeds reveals task-specific performance patterns reflecting the underlying design principles of each method. This finding aligns with recent observations in foundation models for biology (Theodoris et al., 2023; Cui et al., 2024; Gong et al., 2023), where task-specific architectures often outperform general-purpose approaches. Topology-focused methods such as Spectral (Belkin and Niyogi, 2002), GCN (Kipf and Welling, 2017), and DeepWalk (Perozzi et al., 2014) achieve strong link prediction performance (AUROC 0.949–0.977) as network structure alone determines edge existence. These methods learn representations that preserve local neighborhood patterns (Hamilton et al., 2017), making them well-suited for predicting missing edges. In contrast, functional clustering requires information beyond network topology (Barabási et al., 2011; Menche et al., 2015) as proteins in the same pathway may be separated by multiple network hops while topologically adjacent proteins may perform entirely different functions. The controlled comparison between RAG-GNN and its GNN-only ablation (the same architecture with and without retrieval integration) demonstrates a consistent silhouette improvement of +0.093 ± 0.022 across all seeds, providing evidence that retrieved knowledge (Lewis et al., 2020; Gao et al., 2023) contributes to functional clustering quality. ARI also improves (+0.021 ± 0.015), indicating that contrastive alignment during joint fine-tuning improves both intra-cluster cohesion and pairwise cluster agreement, while NMI remains comparable between the two configurations. The heuristic information decomposition (Williams and Beer, 2010) reveals that topology and retrieval encode overwhelmingly shared information (shared component = 95.6%), with minimal unique contributions from either source (topology: 0.1%, retrieval: 6.2%) and negligible synergy (0.4%). The functional clustering improvements arise not from unique retrieval information but from how the fusion mechanism reorganizes shared information to improve intra-cluster cohesion. These findings establish that RAG integration provides measurable benefit for functional interpretation within a controlled experimental framework, suggesting a principled approach: use topology-focused methods for structural tasks and consider RAG-enhanced methods when functional interpretation is the primary objective.

The temporal therapeutic-target evaluation in Section 4.2 should be read in the same controlled spirit. With only three post-2020 FDA-approved therapeutic targets inside the 379-protein cancer signaling network, the temporal AUROC (0.450 ± 0.088; 95% CI [0.301, 0.544]) is inconclusive: The point estimate sits below chance and the confidence interval spans both chance-level performance and values below chance, but the test set is far too small to support either a positive prospective-discovery claim or a strong negative claim that RAG-GNN cannot identify future targets. Consistent with the shuffled-document control and the information-theoretic decomposition discussed in the preceding paragraph, we do not interpret this number as evidence about precision-medicine utility on the present case study. The appropriate falsifiable expectation is that scaling to genome-wide PPI networks and substituting pretrained biomedical encoders should yield tens of post-2020 annotated test targets and a temporal AUROC whose confidence interval is bounded away from chance; that prospective evaluation is deferred to future work (Section 10), and the precision-medicine and drug-response formulations of Section 5 are correspondingly treated as illustrative downstream task formulations rather than empirically supported claims of the present study.

Several limitations constrain current capabilities. The case study uses a 379-protein cancer signaling network, a moderately sized system that limits statistical power for tasks requiring large test sets; temporal therapeutic target prediction, for instance, yields AUROC = 0.450 ± 0.088 with only 3 post-2020 test targets. Scaling to whole-genome networks (>20,000 genes) is necessary for clinically meaningful temporal evaluation but remains computationally challenging (Zeng et al., 2020). The counterfactual experiments reveal that random vectors degrade performance (Δ_random_ = 0.16), confirming that the model depends on real document content rather than additional input dimensionality. As discussed above, the shuffled-document control implies that the current TF-IDF corpus carries general biological signal in its aggregate term statistics; larger corpora and pretrained biomedical encoders may sharpen the distinction. Node features are limited to three topological properties (degree, clustering coefficient, and betweenness centrality); incorporating protein language model embeddings (Rives et al., 2021; Lin et al., 2023) or multi-omics data would strengthen the input representation. More broadly, retrieval quality depends on corpus comprehensiveness (Gao et al., 2023; Zhang et al., 2024); rare diseases with limited literature benefit less from RAG augmentation. The current formulation treats networks as static, ignoring temporal dynamics during disease progression. Current predictions identify correlations rather than causation (Ideker and Krogan, 2012). While retrieved documents provide some interpretability, the gated fusion mechanism offers limited insight into how individual retrieved passages influence predictions, a limitation shared by attention-based architectures (Vaswani et al., 2017). Taken together, these constraints (moderate network size, TF-IDF document features, the shuffled-document control, and the wide-confidence-interval temporal evaluation) mean that the present results should be read as a controlled methodological study on a single PPI-scale case, not as evidence that RAG-GNN is ready for precision-medicine deployment or prospective target discovery; we therefore restrict our claims to method-level behavior under this experimental protocol. A natural next step, identified explicitly by the shuffled-document control, is to replace the TF-IDF document encoder with pretrained biomedical language models such as PubMedBERT (Gu et al., 2022; Hays and Richardson, 2026) and to scale the document corpus from curated mechanistic annotation templates to time-stamped PubMed abstracts; this is the experimental configuration under which we would expect Δ_shuffled_ < Δ_proper_ to become measurable if node-specific semantic matching genuinely contributes beyond aggregate corpus term statistics.

A second scale-level constraint, distinct from but compounding the above, is that all empirical conclusions of this study rest on a single 379-protein cancer signaling network. The runtime and FAISS-indexing measurements (Section 6.1 and Appendix A.8) establish computational feasibility but do not by themselves establish that the modest silhouette and ARI gains observed here would persist on a genome-scale interactome (>20,000 nodes), where the annotation corpus is noisier, pathway memberships overlap more heavily, and the matched GNN-only baseline may itself shift in absolute performance. The shuffled-document control and the wide-CI temporal AUROC reported above therefore characterize a particular PPI-scale operating point, and we cannot claim that the same +0.093 ± 0.022 silhouette improvement would transfer to whole-genome networks without re-running the controlled ablation at that scale. The framework has not been validated on larger biological networks in this study, and the observed effects may not generalize; cross-network replication on genome-scale PPI and on non-cancer pathway systems is therefore stated explicitly as a prerequisite for any future generalization claim, and we provide method-level evidence on one PPI-scale case.

Future directions include extensions to temporal networks through recurrent updates ${\text{z}}_{i}\left(t\right)=f_{\text{temporal}}\left({\text{h}}_{i}^{\left(L\right)}\left(t\right),{\text{c}}_{i}\left(t\right),{\text{z}}_{i}\left(t-\Delta t\right)\right)$ where *t* indexes time points, with time-aware retrieval prioritizing recent publications. Multi-modal integration could extend the contrastive objective across modalities (network, image, EHR, and genomic) to learn aligned representations (Velez-Arce et al., 2024; Huang et al., 2021). Recent advances in geometric graph neural networks for multi-omics data integration (Ramirez et al., 2023) and prior knowledge-guided multilevel GNN frameworks (Yan et al., 2024) demonstrate the potential for combining our RAG-enhanced embeddings with heterogeneous biological data types including transcriptomics, proteomics, lipidomics, nutrigenomics, and metabolomics, enabling more comprehensive patient stratification and biomarker discovery. Incorporating causal inference methods (Pearl, 2009) could enable interventional predictions by estimating causal effects τ_*i*_ = 𝔼[*Y* ∣ *do*(*v*_*i*_ = 0)] − 𝔼[*Y* ∣ *do*(*v*_*i*_ = 1)] using propensity score weighting or instrumental variables. For clinical adoption, natural language explanations generated by prompting large language models with retrieved documents, counterfactual analysis identifying minimal changes that alter predictions, and enhanced attention visualizations (Veličković et al., 2018) could improve interpretability.

## 11 Conclusion

This study establishes mathematical and empirical foundations for integrating retrieval-augmented generation with biological network modeling. We developed joint optimization objectives that simultaneously train network encoders, dense retrievers, and fusion mechanisms through contrastive learning with formal generalization bounds, including proof of retrieval consistency under Lipschitz continuity and geometric characterization of embedding space convergence (Appendix B). The end-to-end trainable RAG-GNN implementation demonstrates consistent improvement in functional clustering: Silhouette score improves from −0.237 ± 0.065 (GNN-only) to −0.144 ± 0.066 (+0.093 ± 0.022) across 10 random seeds, with ARI also improving (+0.021 ± 0.015), while the learned retrieval projection achieves mean precision at 10 = 0.242, a 152% improvement over the random baseline. Heuristic information decomposition reveals that topology and retrieval encode overwhelmingly shared information (95.6% shared), with minimal unique contributions from either source and negligible synergy. The functional clustering improvements arise from how the fusion mechanism reorganizes shared information to improve intra-cluster cohesion. Counterfactual experiments confirm that adversarial, absent, and random retrieval all degrade performance, validating that the gated fusion mechanism depends on retrieval content. DDR1 subnetwork analysis provides confirmatory validation consistent with established synthetic lethality relationships (Ambrogio et al., 2016; Zhavoronkov et al., 2019).

These findings clarify appropriate use cases: The controlled ablation demonstrates that retrieval integration improves functional clustering within the same architecture, while topology-focused methods achieve superior structural prediction. This complementarity suggests that method selection should be guided by the specific task, rather than assuming universal superiority of either approach. Because all empirical claims in this study rest on a single 379-protein PPI-scale case study, cross-network replication on genome-scale interactomes and on non-cancer pathway systems is a prerequisite for any generalization claim and is the immediate next step identified by the scale-limitations discussion in Section 10. More broadly, by calibrating what retrieval augmentation does and does not contribute when coupled with a GNN over a biomedical interaction network, the study contributes methodological groundwork for AI systems intended to support medical knowledge integration and network-based modeling; empirical validation for any specific downstream medical application is itself deferred to studies at larger network scale and with prospective evaluation.

## Data Availability

Publicly available datasets were analysed in this study. This data can be found here: https://github.com/HasiHays/RAG-GNN.
