# Supplementary material for: RAG-GNN: retrieval-augmented graph neural networks for protein interaction network embeddings
Source: Front Artif Intell. 2026 Jul 10;9:1851917. doi: 10.3389/frai.2026.1851917 (PMC13395942; doi:10.3389/frai.2026.1851917)
Supplement: Supplementary file 1 [file Supplementary_file_1.pdf]

Supplementary Materials:
RAG-GNN: Retrieval-Augmented Graph Neural Networks for Protein Interaction Network Embeddings

Hasi Hays[1,*] and **William J. Richardson**[1]

[1] *Department of Chemical Engineering, University of Arkansas, Fayetteville, AR 72701, USA*
[*] *Correspondence:* hasih@uark.edu

A Supplementary materials

This supplementary section provides detailed mathematical derivations and implementation specifics for the RAG-GNN framework that complement the main text.

A.1 Graph neural network message passing

The GNN encoder implements spectral graph convolutions through iterative neighborhood aggregation. Given adjacency matrix $\mathbf{A} \in \mathbb{R}^{n\times n}$ and initial node features $\mathbf{H}^{(0)} \in \mathbb{R}^{n\times d}$, we first compute the normalized adjacency matrix.

Normalized adjacency computation. Add self-loops and compute symmetric normalization:

$$\tilde{\mathbf{A}} = \mathbf{A} + \mathbf{I}_n \tag{34}$$

$$\tilde{\mathbf{D}}_{ii} = \sum_j \tilde{A}_{ij} \tag{35}$$

$$\hat{\mathbf{A}} = \tilde{\mathbf{D}}^{-1/2}\tilde{\mathbf{A}}\tilde{\mathbf{D}}^{-1/2} \tag{36}$$

The symmetric normalization in Eq. 36 ensures that the spectral radius of $\hat{\mathbf{A}}$ is bounded by 1, preventing numerical instability during deep message passing.

Layer-wise propagation. For layer $\ell \in \{1, \ldots, L\}$:

$$\mathbf{H}^{(\ell)} = \sigma\left(\hat{\mathbf{A}}\mathbf{H}^{(\ell-1)}\mathbf{W}^{(\ell)}\right) \tag{37}$$

where $\mathbf{W}^{(\ell)} \in \mathbb{R}^{d_{\ell-1}\times d_\ell}$ are learnable weights and $\sigma(\cdot)$ is a non-linearity (GELU in our implementation).

Layer normalization. After each layer, we apply layer normalization to stabilize training:

$$\mathbf{h}_i^{(\ell)} \leftarrow \frac{\mathbf{h}_i^{(\ell)} - \mu_i}{\sigma_i + \epsilon} \tag{38}$$

where $\mu_i = \frac{1}{d}\sum_j h_{ij}^{(\ell)}$ and $\sigma_i = \sqrt{\frac{1}{d}\sum_j (h_{ij}^{(\ell)} - \mu_i)^2}$.

A.2 Baseline embedding methods

We provide mathematical formulations for all baseline methods used in benchmarking.

Spectral embedding. Compute the k largest singular vectors of the adjacency matrix:

$$\mathbf{A} \approx \mathbf{U}_k\boldsymbol{\Sigma}_k\mathbf{V}_k^\top \tag{39}$$

The embedding is $\mathbf{Z}_{\text{spectral}} = \mathbf{U}_k\boldsymbol{\Sigma}_k$.

DeepWalk. Approximate random walk co-occurrence through powers of the transition matrix $\mathbf{P} = \mathbf{D}^{-1}\mathbf{A}$:

$$\mathbf{M}_{\text{DW}} = \mathbf{P} + \mathbf{P}^2 + \mathbf{P}^3 \tag{40}$$

Apply truncated SVD to obtain embeddings: $\mathbf{Z}_{\text{DW}} = \text{SVD}_k(\mathbf{M}_{\text{DW}})$.

Node2Vec. Combine different random walk orders with biased weighting:

$$\mathbf{M}_{\text{N2V}} = 0.5\mathbf{P} + 0.3\mathbf{P}^2 + 0.2\mathbf{P}^3 \tag{41}$$

The coefficients simulate the effect of return parameter p and in-out parameter q controlling walk behavior.

LINE. Preserve first-order (direct) and second-order (shared neighbor) proximity:

$$\mathbf{M}_{\text{LINE}} = 0.5\mathbf{A} + 0.5\mathbf{A}^2 \tag{42}$$

GCN. Three-layer graph convolutional network:

$$\mathbf{H}^{(1)} = \tanh(\hat{\mathbf{A}}\mathbf{H}^{(0)}) \tag{43}$$

$$\mathbf{H}^{(2)} = \tanh(\hat{\mathbf{A}}\mathbf{H}^{(1)}) \tag{44}$$

where $\mathbf{H}^{(0)} \sim \mathcal{N}(0, 1)$ provides random initialization.

GraphSAGE. Concatenate self-features with aggregated neighbor features:

$$\mathbf{Z}_{\text{SAGE}} = \text{SVD}_k\left([\mathbf{H}^{(0)} \| \mathbf{D}^{-1}\mathbf{A}\mathbf{H}^{(0)}]\right) \tag{45}$$

GAT. Attention-weighted aggregation using softmax over neighbor scores:

$$\alpha_{ij} = \frac{\exp(\mathbf{a}^\top[\mathbf{W}\mathbf{h}_i \| \mathbf{W}\mathbf{h}_j])}{\sum_{k\in\mathcal{N}(i)} \exp(\mathbf{a}^\top[\mathbf{W}\mathbf{h}_i \| \mathbf{W}\mathbf{h}_k])} \tag{46}$$

A.3 RAG-GNN fusion mechanism

The RAG-GNN framework fuses GNN topology embeddings with retrieved document features through the following procedure.

Document embedding. Create TF-IDF representations of the document corpus:

$$\mathbf{E}_{\text{doc}} = \text{TF-IDF}(\mathcal{D}) \in \mathbb{R}^{|\mathcal{D}| \times d_{\text{vocab}}} \tag{47}$$

Retrieval scoring. Compute neighborhood-aware retrieval scores:

$$\mathbf{S} = \hat{\mathbf{A}}^2 \mathbf{R} \tag{48}$$

where $\mathbf{R} \in \mathbb{R}^{n \times |\mathcal{D}|}$ contains base relevance scores between proteins and documents.

Top-k retrieval. For each node i, select documents with highest scores:

$$\mathcal{D}_i^{(k)} = \text{argtop}_k(\mathbf{S}_{i,:}) \tag{49}$$

Retrieved feature aggregation. Compute mean of retrieved document embeddings:

$$\mathbf{r}_i = \frac{1}{k} \sum_{j \in \mathcal{D}_i^{(k)}} \mathbf{E}_{\text{doc},j} \tag{50}$$

Weighted fusion. Combine GNN and retrieved representations:

$$\mathbf{z}_i^{\text{fused}} = [\alpha \cdot \mathbf{h}_i^{(L)} \| (1-\alpha) \cdot \mathbf{r}_i] \tag{51}$$

where $\alpha = 0.6$ weights topology features.

Dimensionality reduction. Apply truncated SVD to obtain final embeddings:

$$\mathbf{Z}_{\text{RAG}} = \text{SVD}_d(\mathbf{Z}^{\text{fused}}) \tag{52}$$

A.4 Algorithm pseudocode

Algorithm 1 provides pseudocode for the complete RAG-GNN embedding procedure, consolidating the mathematical formulations into an algorithmic representation.

Algorithm 1 Learnable RAG-GNN Embedding with Curriculum Training

Require: Adjacency matrix $\mathbf{A}$, node features $\mathbf{X}$, document corpus $\mathcal{D}$, layers $L = 3$, retrieval depth $k = 10$
Ensure: Trained model parameters, node embeddings $\mathbf{Z}$

// Preprocessing
$\hat{\mathbf{A}} \leftarrow \tilde{\mathbf{D}}^{-1/2}(\mathbf{A} + \mathbf{I}_n)\tilde{\mathbf{D}}^{-1/2}$ ▷ Normalized adjacency
$\mathbf{E}_{\text{doc}} \leftarrow \text{SVD}_{d_{\text{doc}}}(\text{TF-IDF}(\mathcal{D}))$ ▷ Document embeddings

// Phase 1: GNN pre-training (link prediction)
for epoch $= 1$ to 80 **do**
  $\mathbf{H}^{(0)} \leftarrow \mathbf{X}$
  for $\ell = 1$ to L **do**
    $\mathbf{H}^{(\ell)} \leftarrow \text{ReLU}(\hat{\mathbf{A}}\mathbf{H}^{(\ell-1)}\mathbf{W}^{(\ell)})$
  end for
  Minimize $\mathcal{L}_{\text{task}}$ (link prediction BCE)
end for

// Phase 2: Retrieval projection training
for epoch $= 1$ to 100 **do**
  $\mathbf{q}_i \leftarrow f_{\text{proj}}(\mathbf{h}_i^{(L)})$ ▷ Learned MLP projection
  $\mathcal{D}_i^{(k)} \leftarrow \text{argtop}_k(\mathbf{q}_i \cdot \mathbf{E}_{\text{doc}}^\top)$
  Minimize $\mathcal{L}_{\text{retrieval}} + \lambda_c \mathcal{L}_{\text{contrastive}}$
end for

// Phase 3: Joint fine-tuning
for epoch $= 1$ to 80 **do**
  $\mathbf{r}_i \leftarrow \frac{1}{k}\sum_{j \in \mathcal{D}_i^{(k)}} \mathbf{E}_{\text{doc},j}$ ▷ Retrieved context
  $g_i \leftarrow \sigma(\mathbf{w}_g^\top [\mathbf{h}_i^{(L)} \| \mathbf{r}_i])$ ▷ Learned gate
  $\mathbf{z}_i \leftarrow g_i \cdot \mathbf{h}_i^{(L)} + (1 - g_i) \cdot \mathbf{W}_r \mathbf{r}_i$ ▷ Gated fusion
  Minimize $\mathcal{L}_{\text{task}} + \lambda_r \mathcal{L}_{\text{retrieval}} + \lambda_c \mathcal{L}_{\text{contrastive}}$
end for
return $\mathbf{Z} = \{\mathbf{z}_i\}_{i=1}^{|\mathcal{V}|}$

1.5 Evaluation metrics

Silhouette score. For node i with cluster label c_i:

$$a_i = \frac{1}{|C_{c_i}| - 1} \sum_{j \in C_{c_i}, j \neq i} \|\mathbf{z}_i - \mathbf{z}_j\|_2 \tag{53}$$

$$b_i = \min_{c \neq c_i} \frac{1}{|C_c|} \sum_{j \in C_c} \|\mathbf{z}_i - \mathbf{z}_j\|_2 \tag{54}$$

$$s_i = \frac{b_i - a_i}{\max(a_i, b_i)} \tag{55}$$

The overall silhouette score is $\bar{s} = \frac{1}{n} \sum_i s_i$, ranging from -1 (poor clustering) to $+1$ (perfect clustering).

Link prediction (LP). Generate positive edges $\mathcal{E}^+$ from observed interactions and negative edges $\mathcal{E}^-$ by random sampling non-edges. Prediction scores:

$$\hat{y}_{ij} = \sigma(\mathbf{z}_i^\top \mathbf{z}_j) \tag{56}$$

where σ is the sigmoid function.

Area Under ROC Curve (AUROC). The Receiver Operating Characteristic (ROC) curve plots true positive rate (TPR) against false positive rate (FPR) at varying classification thresholds:

$$\text{TPR} = \frac{\text{TP}}{\text{TP} + \text{FN}}, \quad \text{FPR} = \frac{\text{FP}}{\text{FP} + \text{TN}} \tag{57}$$

AUROC measures the probability that a randomly chosen positive example ranks higher than a randomly chosen negative example. Values range from 0.5 (random) to 1.0 (perfect discrimination).

Area Under Precision-Recall Curve (AUPRC). The Precision-Recall curve plots precision against recall:

$$\text{Precision} = \frac{\text{TP}}{\text{TP} + \text{FP}}, \quad \text{Recall} = \frac{\text{TP}}{\text{TP} + \text{FN}} \tag{58}$$

AUPRC is particularly informative for imbalanced datasets where negative examples dominate, as it focuses on positive class performance without being influenced by true negatives.

Node classification (NC). To avoid information leakage, we construct topology-derived labels independent of functional categories:

$$y_i^{\text{hub}} = \mathbb{1}[d_i > \bar{d} + \sigma_d] \tag{59}$$

$$y_i^{\text{bridge}} = \mathbb{1}[b_i > \text{median}(b)] \wedge \mathbb{1}[c_i < \text{median}(c)] \tag{60}$$

$$y_i^{\text{fair}} = y_i^{\text{hub}} \oplus y_i^{\text{bridge}} \tag{61}$$

where d_i is degree, b_i is betweenness centrality, c_i is clustering coefficient, and $\oplus$ denotes XOR. Node classification performance (NC AUROC) is evaluated by training a logistic regression classifier on node embeddings to predict y_i^{fair}, reporting AUROC on held-out test nodes via 5-fold cross-validation.

1.6 Network statistics

The cancer signaling network from STRING database exhibits the following properties:

- **Nodes:** $n = 379$ proteins
- **Edges:** $m = 3,498$ interactions
- **Average degree:** $\bar{d} = 18.46$
- **Average clustering coefficient:** $\bar{c} = 0.596$
- **Network density:** $\rho = 2m/(n(n-1)) = 0.049$
- **Functional categories:** 14 pathways

The high clustering coefficient (0.596) indicates modular organization typical of biological networks, while the relatively high average degree (18.46) reflects the interconnected nature of cancer signaling pathways.

1.7 Hyperparameter settings

GNN architecture:

- Number of layers: $L = 3$
- Hidden dimension: $d_h = 128$
- Activation: GELU with dropout = 0.1 (retrieval projection)
- Node features: log-degree, clustering coefficient, and scaled betweenness centrality ($d_{\text{input}} = 3$ informative features in d_h-dimensional vector)

Retrieval parameters:

- Documents retrieved per node: $k = 10$
- Document embedding: TF-IDF (256 features, unigrams/bigrams) $\to$ truncated SVD to $d_{\text{doc}} = 64$
- Retrieval projection: two-layer MLP ($d_h \to d_h \to d_{\text{doc}}$, GELU activation)
- Fusion: learned gated mechanism (mean gate $\approx$ 0.593, 59% topology / 41% retrieval)

Training:

- Phase 1 (GNN pre-training): 80 epochs, link prediction loss, lr = 0.003
- Phase 2 (retrieval training): 100 epochs, margin ranking + contrastive loss, lr = 0.005
- Phase 3 (joint fine-tuning): 80 epochs, combined loss, lr = 0.001
- Optimizer: Adam with weight decay = 10^{-4}

Evaluation:

- Link prediction: 20% test edges, negative sampling
- Functional clustering: silhouette score, NMI, ARI with k-means ($k = 14$ categories)
- Random seeds: 10 seeds (42–51) with mean $\pm$ std and 95% bootstrap CIs

1.8 Computational requirements

All experiments were conducted on a single workstation with the following specifications:

- CPU: Apple M-series (Apple silicon M1 or later)
- RAM: 16 GB minimum
- Python: 3.9+
- Key libraries: PyTorch, NumPy, SciPy, NetworkX, scikit-learn

The complete experimental pipeline, including RAG-GNN training across 10 seeds (three phases: 80+100+80 epochs each), eight baseline methods, information decomposition (200 bootstrap resamples), and counterfactual experiments, completes in approximately 88 seconds on an Apple M-series laptop. No GPU acceleration is required for the 379-node network.

2 Formal theoretical framework

This appendix collects the formal theoretical development of the RAG embedding framework that supports the operator definitions in Section 2 and the optimization objectives in Section 3 of the main text. It contains (i) the partial-information decomposition used to quantify the contribution of retrieved documents beyond network topology and its empirical instantiation on the cancer network, (ii) the embedding-space geometry and alignment analysis under the contrastive objective, (iii) PAC-style generalization bounds for link prediction, and (iv) a Lipschitz stability bound for the learned retrieval scores. The three main formal statements appear as Propositions B.1–B.3. These results are formal underpinnings of the framework, not benchmark claims; the empirical evaluation is reported in the main text.

2.1 Information-theoretic decomposition

To quantify the unique information contributed by retrieved documents, we decompose the mutual information between final embeddings $\mathbf{z}_i$ and prediction targets y using the partial information decomposition framework[1]. Define three information sources: network topology $\mathcal{G}$, node features $\mathbf{x}$, and retrieved documents $\mathcal{D}_i^{(k)}$. The total predictive information can be decomposed as:

$$\begin{aligned} I(\mathbf{z}_i; y) = & I_{\text{unique}}(\mathcal{G}) + I_{\text{unique}}(\mathcal{D}_i^{(k)}) \\ & + I_{\text{shared}}(\mathcal{G}, \mathcal{D}_i^{(k)}) \\ & + I_{\text{synergy}}(\mathcal{G}, \mathcal{D}_i^{(k)}) \end{aligned} \tag{62}$$

where $I_{\text{unique}}(\mathcal{G})$ quantifies information provided exclusively by network structure, $I_{\text{unique}}(\mathcal{D}_i^{(k)})$ measures unique contribution from retrieved documents, I_{shared} captures redundant information present in both sources, and I_{synergy} represents emergent information available only when both sources are combined. We estimate these quantities using a heuristic minimum-redundancy decomposition where shared information is estimated as $\min(I_{\text{gnn}}, I_{\text{ctx}})$. The key metric is the normalized unique retrieval contribution:

$$\rho_{\text{unique}} = \frac{I_{\text{unique}}(\mathcal{D}_i^{(k)})}{I(\mathbf{z}_i; y)} \tag{63}$$

Non-zero values of ρ_{unique} indicate that retrieved documents contribute predictive information not available from network topology alone. We validate this empirically in our cancer network experiments (Section 7.4) using 200 bootstrap resamples. The heuristic decomposition reveals that topology and retrieval encode overwhelmingly shared information (shared component = 95.6%), with minimal unique contributions from either source (topology: 0.1%, retrieval: 6.2%) and negligible synergy (0.4%). This high shared component indicates that the contrastive alignment during joint training effectively coordinates topology and retrieval representations into overlapping information spaces. The functional clustering improvements observed in Section 7.2 arise not from unique retrieval information, but from how the fusion mechanism reorganizes shared information to improve intra-cluster cohesion.

2.2 Embedding-space geometry and alignment

The joint embedding space exhibits geometric properties that reflect both network topology and semantic relationships. Define the structural similarity between nodes v_i and v_j based on network proximity:

$$S_{\text{struct}}(v_i, v_j) = \mathbf{h}_i^{(L)\top} \mathbf{h}_j^{(L)} \tag{64}$$

and semantic similarity based on document embeddings:

$$S_{\text{sem}}(v_i, v_j) = E_{\text{node}}(v_i)^\top E_{\text{node}}(v_j) \tag{65}$$

The alignment between structural and semantic similarity quantifies how well the embedding space integrates both information sources:

$$\rho_{\text{align}} = \text{cor}\left(\{S_{\text{struct}}(v_i, v_j)\}, \{S_{\text{sem}}(v_i, v_j)\}\right) \tag{66}$$

where the correlation is computed over all node pairs.

Proposition B.1 (Embedding alignment). Under the contrastive loss in **??** with temperature τ, the embedding functions E_{node} and E_{doc} converge to representations where $\rho_{\text{align}} \geq 1 - \delta$ for any $\delta > 0$ as the number of training iterations $t \to \infty$, provided: (i) the temperature $\tau < 1/\log|\mathcal{D}|$, (ii) node-document associations are consistent, and (iii) the learning rate schedule satisfies $\sum_{t=1}^{\infty} \eta_t = \infty$ and $\sum_{t=1}^{\infty} \eta_t^2 < \infty$.

Proof sketch. The contrastive objective maximizes the inner product $E_{\text{node}}(v_i)^\top E_{\text{doc}}(d_i^+)$ for associated pairs while minimizing inner products with negative documents. In the limit $\tau \to 0$, this corresponds to hard negative mining where only the most similar negative document contributes gradient signal. The InfoNCE loss[2] provides a lower bound on mutual information:

$$I(E_{\text{node}}(V); E_{\text{doc}}(D^+)) \geq \log|\mathcal{D}| - \mathcal{L}_{\text{contrastive}} \tag{67}$$

Maximizing this bound drives the embeddings to encode shared information between nodes and documents. Under the Robbins-Monro conditions on learning rates, stochastic gradient descent converges to a critical point where gradients vanish, implying high correlation between structural and semantic similarities. The consistency assumption ensures that nodes with similar network positions have semantically related documents, enabling alignment.

2.3 Generalization bounds for link prediction

For the link prediction task, we derive PAC-style generalization bounds relating training and test performance. Let

$\mathcal{H}$ denote the hypothesis class of RAG-GNN models with bounded parameter norm $\|\theta\|_2 \leq B$, and let $n = |\mathcal{E}^+|$ be the number of positive training edges.

Proposition B.2 (Generalization bound). With probability at least $1-\delta$ over the random selection of training edges, for any hypothesis $h \in \mathcal{H}$ with parameters θ, the true risk satisfies:

$$\mathcal{L}_{\text{true}}(h) \leq \mathcal{L}_{\text{train}}(h) + \mathcal{O}\left(\sqrt{\frac{B^2 d_z \log(|\mathcal{V}|/\delta)}{n}}\right) + \epsilon_{\text{retrieval}} \tag{68}$$

where $\mathcal{L}_{\text{true}}$ is the expected loss on the true distribution of edges, $\mathcal{L}_{\text{train}}$ is the empirical training loss, and $\epsilon_{\text{retrieval}} = \mathcal{O}(k/|\mathcal{D}|)$ accounts for retrieval approximation error.

Proof sketch. The bound follows from Rademacher complexity analysis of the hypothesis class. The link prediction function $f(v_i, v_j) = \sigma(\mathbf{z}_i^\top \mathbf{z}_j)$ has Lipschitz constant $L_f \leq B^2$ with respect to edge labels. The Rademacher complexity of linear functions over embedding spaces with bounded norm is:

$$\mathfrak{R}_n(\mathcal{H}) \leq \frac{B\sqrt{d_z}}{\sqrt{n}} \tag{69}$$

Applying standard uniform convergence results[3] with union bound over all nodes yields the first term. The retrieval error arises because approximate top-k retrieval may miss relevant documents, bounded by the fraction of documents retrieved relative to corpus size. This bound reveals that generalization improves with more training edges (n), lower model complexity (smaller B and d_z), and higher retrieval accuracy (larger k or more focused corpus). Notably, the bound depends on embedding dimension d_z rather than raw network size $|\mathcal{V}|$, showing that learned representations provide effective dimensionality reduction.

2.4 Retrieval consistency and stability

An important property for practical deployment is retrieval stability: small perturbations to node features should not drastically alter retrieved documents. Define the retrieval consistency as:

$$\text{Consistency}(\epsilon) = \mathbb{P}\left[\mathcal{D}_i^{(k)} = \mathcal{D}_{i'}^{(k)} \mid \|\mathbf{x}_i - \mathbf{x}_{i'}\|_2 < \epsilon\right] \tag{70}$$

measuring the probability that nodes with similar features retrieve identical document sets.

Proposition B.3 (Retrieval stability bound). If the node embedding function E_{node} is L_E-Lipschitz continuous, then for any two nodes $v_i, v_{i'}$ with feature perturbation $\|\mathbf{x}_i - \mathbf{x}_{i'}\|_2 \leq \epsilon$:

$$|R(v_i, d_j) - R(v_{i'}, d_j)| \leq \frac{L_E \epsilon \|E_{\text{doc}}(d_j)\|_2 Q(d_j)}{\sqrt{d_e}} \tag{71}$$

for any document $d_j \in \mathcal{D}$.

Proof. By Lipschitz continuity of E_{node}:

$$\|E_{\text{node}}(v_i) - E_{\text{node}}(v_{i'})\|_2 \leq L_E \|\mathbf{x}_i - \mathbf{x}_{i'}\|_2 \leq L_E \epsilon \tag{72}$$

The retrieval score difference is:

$$|R(v_i, d_j) - R(v_{i'}, d_j)| \tag{73}$$

$$= \left| \frac{E_{\text{node}}(v_i)^\top E_{\text{doc}}(d_j) Q(d_j) - E_{\text{node}}(v_{i'})^\top E_{\text{doc}}(d_j) Q(d_j)}{\sqrt{d_e}} \right| \tag{74}$$

$$= \left| \frac{(E_{\text{node}}(v_i) - E_{\text{node}}(v_{i'}))^\top E_{\text{doc}}(d_j) Q(d_j)}{\sqrt{d_e}} \right| \tag{75}$$

$$\leq \frac{\|E_{\text{node}}(v_i) - E_{\text{node}}(v_{i'})\|_2 \|E_{\text{doc}}(d_j)\|_2 Q(d_j)}{\sqrt{d_e}} \tag{76}$$

$$\leq \frac{L_E \epsilon \|E_{\text{doc}}(d_j)\|_2 Q(d_j)}{\sqrt{d_e}} \tag{77}$$

by the Cauchy–Schwarz inequality.

Remark. Proposition B.3 applies the classical Lipschitz continuity framework, a well-established concept from real analysis, to derive stability guarantees specific to the RAG-GNN architecture. The contribution is not the Lipschitz property itself, but rather: (i) proving that the composed retrieval score function $R(v, d)$ inherits Lipschitz stability from the node encoder, (ii) deriving the explicit dependence on document embeddings $\|E_{\text{doc}}(d_j)\|_2$, quality scores $Q(d_j)$, and embedding dimension d_e, and (iii) connecting the bound to practical regularization strategies for graph neural networks. The Lipschitz constant L_E is bounded by the product of spectral norms of GNN weight matrices: $L_E \leq \prod_{k=1}^{L} \sigma_{\max}(\mathbf{W}^{(k)})$. Regularizing weight matrices through spectral normalization ensures small L_E, providing stable retrieval. This stability is relevant for clinical applications where small measurement noise should not radically alter therapeutic recommendations.

2.5 Data availability

Cancer network data are obtained from the Cancer Gene Census (https://cancer.sanger.ac.uk/census; 379 cancer-associated proteins) and the STRING database (https://string-db.org; 3,498 protein-protein interactions filtered at combined score > 400). The document corpus consists of 1,895 synthetic mechanistic-annotation templates generated programmatically from 14 functional pathway categories (see `examples/learnable_cancer_network.py` in the code repository). Processed datasets, including the protein network, functional annotations, and complete code for document corpus generation, are available in the GitHub repository. Detailed mathematical derivations, hyperparameter settings, and computational requirements are provided in Section A.

2.6 Code availability

The RAG-GNN framework implementation is publicly available at https://github.com/HasiHays/RAG-GNN. The repository includes source code, example scripts, documentation, and instructions for reproducing the results presented in this manuscript.

References

[1] Paul L Williams and Randall D Beer. Nonnegative decomposition of multivariate information. *arXiv preprint arXiv:1004.2515*, 2010. URL https://arxiv.org/abs/1004.2515.

[2] Aaron van den Oord, Yazhe Li, and Oriol Vinyals. Representation learning with contrastive predictive coding. *arXiv preprint arXiv:1807.03748*, 2018. URL https://arxiv.org/abs/1807.03748.

[3] Peter L Bartlett and Shahar Mendelson. Rademacher and gaussian complexities: Risk bounds and structural results. *Journal of Machine Learning Research*, 3:463–482, 2002.
